# The good, the bad, and the ugly: Compliance of e-pharmacies serving India and Kenya with regulatory requirements and best practices

**DOI:** 10.1371/journal.pgph.0004202

**Published:** 2025-02-03

**Authors:** Gautam Satheesh, Sammy Masibo, Sasi Kumar Tiruttani, Irene Khayoni, Benjamin Palafox, Devaki Nambiar, Jaison Joseph, Emmanuel Kweyu, Abdul Salam, Francis Wafula, Catherine Goodman

**Affiliations:** 1 The George Institute for Global Health, Hyderabad, India; 2 Strathmore Business School, Strathmore University, Nairobi, Kenya; 3 Department of Global Health and Development, London School of Hygiene & Tropical Medicine, London, United Kingdom; 4 The George Institute for Global Health, New Delhi, India; 5 Faculty of Medicine, University of New South Wales, Sydney, Australia; 6 Prasanna School of Public Health, Manipal Academy of Higher Education, Manipal, India; 7 iLabAfrica, Strathmore University, Nairobi, Kenya; University of Oslo Faculty of Medicine: Universitetet i Oslo Det medisinske fakultet, NORWAY

## Abstract

As with most technology-driven change, e-pharmacy markets have expanded faster than the pace of regulation, particularly in low- and middle-income countries. We developed and applied a checklist to assess compliance with best practices and regulations by e-pharmacies serving clients in India and Kenya, two countries with contrasting regulatory environments. We defined e-pharmacies as businesses selling prescription-only medicines directly to consumers through websites or apps. We identified the universe of e-pharmacies through online searches, and captured data using a structured questionnaire (Jan–May 2023). We then assessed e-pharmacies against a set of global ‘best practice’ standards, as well as national regulations (for Kenya) and ‘proposed requirements’ from local guidelines and draft bills (for India, which had no e-pharmacy-specific regulations). We identified 61 websites and 37 apps serving India, and 26 websites and 3 apps serving Kenya. Regarding best practices, a facility to upload prescriptions was provided by 90% of websites serving India and 58% serving Kenya. Only 16% (India) and 42% (Kenya) provided complete drug information. On average, websites serving Kenya met 8.9 of the 12 (74%) Kenyan regulatory requirements, while those serving India met 7.5 of the 14 (54%) ‘proposed requirements’. Only 31% serving Kenya and none serving India displayed required registration numbers. Contrary to regulations/guidelines, many e-pharmacies serving Kenya (62%) and India (34%) listed narcotic/controlled drugs for sale. In both countries, high-traffic websites and e-pharmacies located within the study country had higher mean compliance to regulation and best practices compared to the others. These findings can be leveraged to strengthen enforcement in Kenya and inform the development of a comprehensive regulatory framework in India. We recommend a risk-based regulatory approach, where regulators work with the largely compliant (“good”) e-pharmacies, improve enforcement among the partially compliant (“bad”), and eliminate the largely non-compliant (“ugly”) from the market.

## Author summary

**Why was this study done?:**

- E-pharmacy markets have been growing rapidly in low- and middle-income countries (LMICs)
- Poorly regulated e-pharmacy can expose consumers to health and e-commerce-related risks.
- There is limited evidence on characteristics of e-pharmacies in LMICs and their compliance to best practices and regulations.

**What did the researchers do and find?:**

- We identified all e-pharmacies serving consumers in India and Kenya, two LMICs with contrasting market sizes and regulatory environments, and assessed compliance to global best practices and existing or proposed national regulations.
- The results highlight low compliance to best practice standards and national regulations/guidelines in both countries.
- Compliance was generally higher for e-pharmacies that had higher-traffic or were located within the country of study.

**What do these findings mean?:**

- The study findings can be leveraged to strengthen regulatory enforcement in Kenya, and inform the development of legislation and accompanying regulations for e-pharmacies in India.
- We recommend a risk-based approach to regulation, where regulators work with the largely compliant (“good”) e-pharmacies, improve enforcement among the partially compliant (“bad”), and eliminate the largely non-compliant (“ugly”).

## Introduction

Access to essential medicines is critical for effective health system performance, yet many low- and middle-income countries (LMICs) continue to face challenges of accessibility, affordability and quality [1]. Online retail pharmacies, or e-pharmacies, once the preserve of high-income countries (HIC), present potential opportunities to address some of these challenges in LMICs, given the convenience and flexibility associated with buying medicines online, particularly for patients with chronic conditions [2].

Over the past decade, the global e-pharmacy market has experienced rapid growth. Valued at USD 92 billion in 2023, it is expected to exceed USD 350 billion by 2033, with emerging markets contributing the larger share [3]. For example, 2–3% of total medicine sales in India are currently through e-pharmacies, a figure expected to rise to 15% over the next decade [4]. Similar growth has been observed in other LMICs, facilitated by rapid internet penetration and COVID-19 pandemic-related movement restrictions. The African e-pharmacy market tripled from a pre-COVID-19 value of USD 190 million in 2019 to USD 560 million in 2022 [5]. While e-pharmacy use is currently likely to be higher among those belonging to higher socioeconomic status or urban populations, given increasing internet penetration, its use among poorer urban and rural communities is also likely to increase.

Regulations to promote pharmacy care and patient safety have not kept pace with this rapid market growth in LMICs [6], exposing consumers to risks like accessing prescription-only medicines (POMs) without prescription, inadequate medicine information, and substandard and/or falsified medicines, alongside other (non-health) risks like compromised data privacy and consumer fraud [7]. There is also the risk of ‘rogue’ e-pharmacies (unlicensed actors that closely resemble legitimate medicine providers, but eschew governing laws and regulations) [8].

The majority of studies on e-pharmacies come from HIC settings [9,10], where the regulatory frameworks and mechanisms for identifying non-compliance are more mature [2,8]. While there is a growing literature on e-pharmacies in LMICs [5,6,11,12], policymakers require better quality and context-specific evidence on e-pharmacy practices and regulatory compliance to design fit-for-purpose governance mechanisms. The aim of this study was to assess the characteristics and business practices of e-pharmacies, and their compliance with global best practice guidelines and national regulatory requirements. We reviewed the websites and apps of all e-pharmacies serving retail customers in two LMICs: India, which has a very large e-pharmacy market, but is yet to enact formal e-pharmacy regulations, and Kenya, which has a smaller market, but with developed regulations.

## Methods

### Eligibility

For the purposes of this study, an e-pharmacy was defined as an online provider that met all the following criteria:

- Sells modern/allopathic POMs directly to consumers in India or Kenya, with or without over-the-counter (OTC) products and/or traditional, complementary, and alternative medicines (TCAM);
- Operates through an internet website or mobile app, through which the ordering process can be initiated (we exclude brick-and-mortar pharmacies with a website for promotional purposes only where it is not possible to initiate an order);
- Offers a mechanism for making payments remotely (e.g., online, cash on delivery);
- Offers delivery through mail, shipping companies, or courier services.

The definition includes both legitimate e-pharmacies operating within local regulations and those operating without a licence and/or contravening other requirements. We also included businesses that met all the criteria but claimed on the website that they were ‘not a pharmacy’, possibly to avoid regulatory scrutiny. For e-pharmacies with websites that direct consumers to their associated app as the sole e-commerce platform, we only reviewed the app. E-pharmacies physically based inside and outside of the study countries were included as long as they served customers within the study country. We excluded marketplace websites which direct consumers to a range of online and/or brick-and-mortar pharmacies, instead reviewing the individual e-pharmacies. While most e-pharmacy websites displayed a catalogue of medicines and other products available for sale, a few did not. These websites were assumed to sell POMs and, therefore, be eligible if they (i) stated that they sold POMs (ii) provided an option to upload a prescription, or (iii) stated that prescriptions were required to complete purchases. Some e-pharmacies had multiple websites, known as ‘clones’ or ‘affiliate networks’ [13]. We identified clones by looking for similar appearance (website templates, offers, and customer testimonials), and then confirmed clone status based on the presence of common contact information (telephone number, business address, or bank account numbers) or if we were redirected to an affiliate website. Where clones were identified, we only reviewed the website that appeared first in our search.

### Search strategy

We aimed to identify the entire population of e-pharmacies retailing medicines to consumers in Kenya and India. To do this we replicated typical consumer internet search behaviour. In each country, separate searches were conducted on the Google search engine using the strings [“India/Kenya AND online pharmacy OR e-pharmacy OR buy medicines online”], [“India/Kenya AND buy antibiotics online without prescription”], and [“India/Kenya AND buy Viagra online”]. Searches were conducted using ‘incognito windows’ so that stored data on past search history (e.g., cookies) did not influence the presentation or ordering of search results. We screened the first 30 pages of search results (or all pages where there were less than 30), and e-pharmacy websites or apps that met our definition were included.

### Data extraction

A structured website review tool and a parallel app review tool were developed on REDCap (Research Electronic Data Capture), a secure web-based platform, hosted at The George Institute for Global Health, India [14]. The review tools comprised a comprehensive checklist covering e-pharmacy characteristics, and adherence to best practices, available national regulations and/or guidelines that could be assessed through website review. The list of best practices was created based on international standards for e-pharmacy, requirements in countries with established regulations, and previous literature on e-pharmacy [2,8,15,16].

While many items in Kenyan and Indian regulations and guidelines overlap with best practices, we also present adherence to country-specific requirements for each country. In Kenya, the regulatory requirements were based on the November 2022 Guidelines for Internet Pharmacy Service and the Guidelines for the Registration and Licensing of Premises in Kenya, which were the most current at the time the study was initiated [17]. These guidelines were updated later in April 2023 to include some additional provisions, which we did not assess [18].

Unlike Kenya, India has not enacted regulations specifically for e-pharmacy. We therefore assessed compliance to a set of ‘proposed requirements’ drawn from available guidelines [19], draft regulations [20,21], and existing applicable regulations not specific to e-pharmacy [22–26]. The two draft regulations, i.e., the Drugs and Cosmetics (Draft) Amendment Rules (2018) [20,21] and Draft Drugs, Cosmetics, and Medical Devices Bill (2022) [20] propose some requirements for e-pharmacy, but have not been enacted as yet. See S1 Table for full details on the documents used to identify the Kenyan regulations and Indian ‘proposed requirements’.

For each included website and app, the review tools captured information related to the following domains: basic characteristics, authorisation details, customer service, privacy, payment, service coverage, extent of drug information provided, and marketing strategies (for complete tool see S1 File). All information extracted from websites and apps was taken at face value, and any claims made by e-pharmacies (e.g., availability of telephone helpline or prescription upload facility) were not tested for authenticity or otherwise verified.

Data extraction was conducted between January and May 2023. In each country, data extraction was done independently and in duplicate by two reviewers. Disagreements were resolved by consensus, consulting a third reviewer where necessary. Websites were reviewed first, and if they claimed to have an app, the corresponding app was downloaded from the Google Play store by both reviewers. As all encountered apps were available as Android versions, and iOS versions were available only for a subset of apps in the Apple App store (see Results), we only reviewed the Android version of the apps. We excluded outdated apps that could not be downloaded because they were no longer compatible with a recent version of Android, and those that were non-functional from the landing page or login stage.

Given the wide range of products stocked by e-pharmacies, data on the availability of information on medicine indications, contraindications, interactions, and side-effects was collected for selected ‘tracer medicines’ used to treat common conditions (amlodipine/nifedipine for hypertension; atenolol/metoprolol for cardiovascular disease; atorvastatin for dyslipidaemia; metformin for diabetes mellitus; amoxicillin/azithromycin for common infections; and sildenafil/tadalafil for erectile dysfunction). The presence of each e-pharmacy on various social media platforms (e.g., Facebook, LinkedIn, Instagram) was assessed by running separate internet searches.

Data on the location of each website’s domain registration and server were obtained through separate searches in each country, using ‘IP Tracker’, a web-based tool used to detect the internet protocol (IP) addresses of websites (www.ip-tracker.org). IP Tracker searches were always done from the respective study countries, as some websites may use servers in multiple locations depending on the user’s location. Data for the number of visits to each website globally over the three-month period (March-May 2023) was obtained from Similarweb.com, an online tool that estimates web traffic volumes (data on visit numbers specifically for the study countries was not consistently available for all websites).

### Data analysis

The data collected from websites and apps were used to produce a range of binary indicators on the presence or absence of a recommended or required feature, which we present as frequencies and percentages. The 12 indicators on compliance with regulations in Kenya and 14 ‘proposed requirements’ in India were used to derive two indices: a binary indicator on whether an e-pharmacy complied with all requirements for that country, presented as frequencies and percentages, and another that sums the number of requirements that an e-pharmacy complied with, presented as the mean across all e-pharmacies.

For websites, we also present these analyses stratified in two ways: (i) based on website visit numbers, comparing the top 20% of websites in terms of visit numbers vs. all other websites; and (ii) based on physical location of the e-pharmacy, comparing those located within vs. outside of the study country.

Data analyses were conducted in Stata 18.0 (StataCorp; College Station, TX) [27]. It was not appropriate to conduct statistical tests for significance because our search strategy sought to identify the entire population of eligible e-pharmacies serving consumers in each study country, i.e., was a census approach, rather than a randomly selected sample.

### Ethics statement

This study was approved by the ethics committees of The George Institute for Global Health (Project number: 26/2022), Strathmore University Business School (Ref. no: SU-ISERC1475/22), and the London School of Hygiene & Tropical Medicine (Ref. no: 28055).

## Results

**Fig 1:**
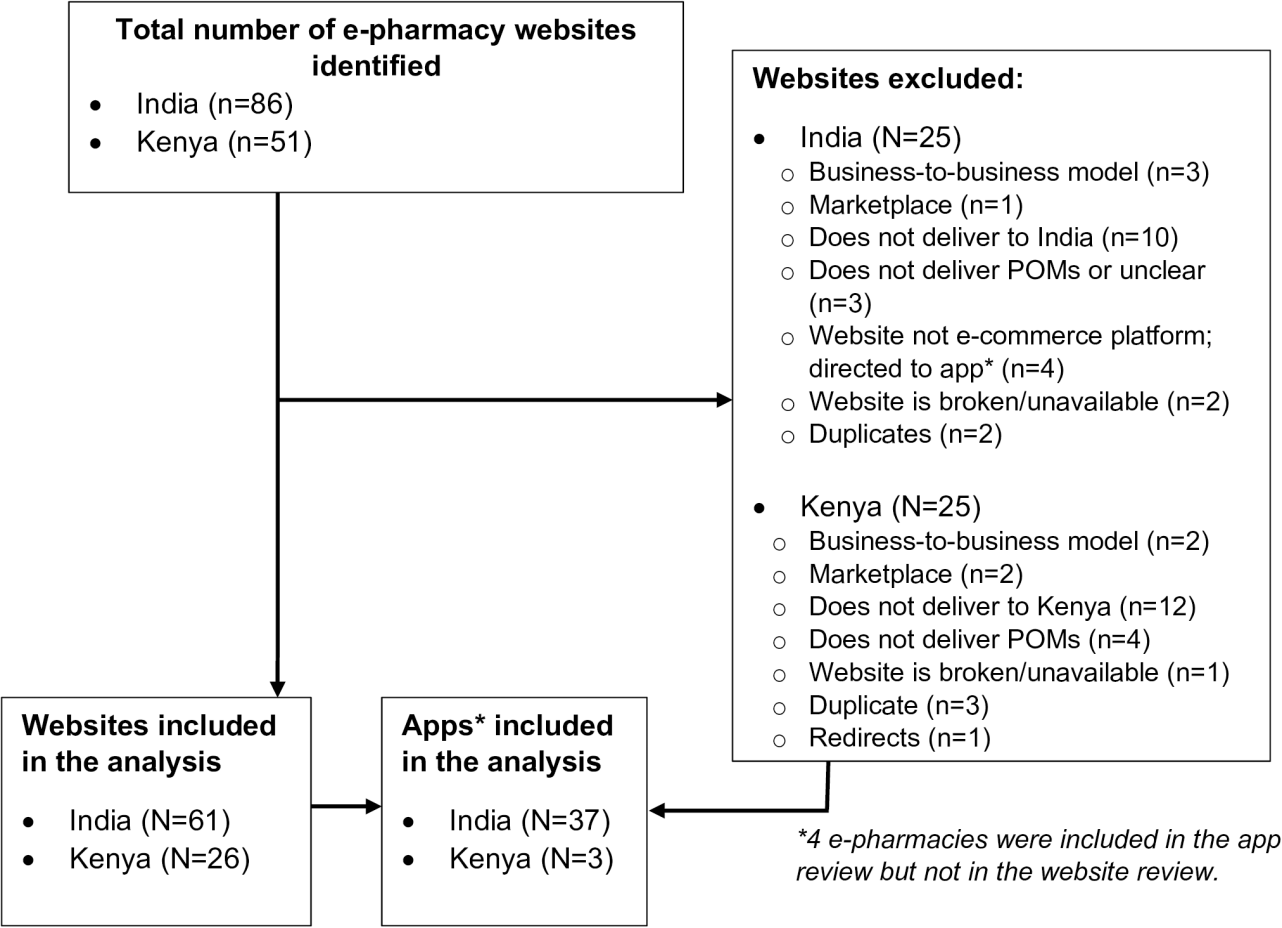
Identification and inclusion of e-pharmacies.

Our search yielded 86 websites serving consumers in India and 51 websites serving consumers in Kenya, of which 61 serving India and 26 serving Kenya met our eligibility criteria and were included in the analysis. See Fig 1. Among included websites, 33 in India and 3 in Kenya had associated apps; additionally, 4 websites serving India (which did not meet our inclusion criteria) directed consumers to their apps, which met the eligibility criteria and were included in the analysis. Only one website served both countries, although it did so using different web addresses.

### E-pharmacy characteristics and business practices

Table 1 summarises the characteristics and business practices of websites and apps. In addition to POMs, all 26 websites serving Kenya also offered OTC medicines and TCAM products, and all but one offered nutraceuticals. These products were also offered by most websites serving India, though not universally. Of the 61 websites serving India, 39% offered online consultations with a doctor, laboratory services, or both, compared with only 15% in Kenya. While for both countries most websites said they had a national or sub-national delivery reach, 21% in India and 31% in Kenya claimed to deliver outside of the study country (geographical reach was unclear for a further 23% in India and 8% in Kenya).

**Table 1:** E-pharmacy characteristics and business practices, n (%).

	India		Kenya	
	Websites	Apps	Websites	Apps
**Number of e-pharmacies reviewed (N)**	**61**	**37**	**26**	**3**
**Products displayed for sale**				
Over-the-counter (OTC) medicines	53 (86.9)	33 (89.2)	26 (100.0)	3 (100.0)
Traditional, complementary and alternative (TCAM) medicines	48 (78.7)	33 (89.2)	26 (100.0)	3 (100.0)
Nutraceuticals	52 (85.2)	34 (91.9)	25 (96.1)	3 (100.0)
**Complementary services provided**				
Online consultation with a doctor	21 (34.4)	18 (48.6)	3 (11.5)	1 (33.3)
Laboratory tests	15 (24.6)	17 (45.9)	2 (7.7)	0 (0.0)
Both	12 (19.7)	13 (35.1)	1 (3.8)	0 (0.0)
Pharmacy only	37 (60.7)	15 (40.5)	22 (84.6)	2 (66.7)
**Coverage of delivery services**				
Delivers only to specific states/counties	20 (32.8)	12 (32.4)	2 (7.7)	1 (33.3)
Delivers anywhere within the country	14 (22.9)	7 (18.9)	14 (53.8)	2 (66.7)
Delivers outside the country	13 (21.3)	1 (2.7)	8 (30.8)	0 (0.0)
Unclear	14 (22.9)	17 (45.9)	2 (7.7)	0 (0.0)
**Availability of apps**				
Android	–	37 (100.0)	–	3 (100.0)
iOS	–	25 (67.6)	–	3 (100.0)
**Country of domain registration**				
Within the study country	28 (45.9)	–	12 (46.2)	–
US	23 (37.7)	–	6 (23.1)	–
Others^1^	9 (14.7)	–	6 (23.1)	–
Unknown	1 (1.6)	–	2 (7.7)	–
**Country of website server**				
Within the study country	23 (37.7)	–	–	–
US	22 (36.1)	–	13 (50.0)	–
Others^2^	6 (9.8)	–	10 (38.5)	–
Unknown	10 (16.4)	–	3 (11.5)	–
**Marketing strategies**				
Announces offers or discounts for medicine^3^	52 (85.2)	30 (81.1)	18 (69.2)	3 (100.0)
Has any social media presence^4^	57 (93.4)	37 (100.0)	21 (80.8)	3 (100.0)
**Customer support/feedback**				
FAQ (Frequently Asked Questions) section	39 (63.9)	21 (56.8)	16 (61.5)	3 (100.0)
Additional language options^5^	8 (13.1)	5 (13.5)	5 (19.2)	0 (0.0)
Customer reviews/testimonials	27 (44.3)	6 (16.2)	21 (80.8)	1 (33.3)
**Payment modes**				
Credit/Debit card	45 (73.8)	24 (64.9)	10 (38.5)	1 (33.3)
Net banking	38 (62.3)	27 (73.0)	3 (11.5)	1 (33.3)
Store credit	6 (9.8)	5 (13.5)	1 (3.8)	0 (0.0)
UPI (Unified Payments Interface)	38 (62.3)	26 (70.3)	0 (0.0)	0 (0.0)
Electronic wallet	30 (49.2)	21 (56.8)	3 (11.5)	0 (0.0)
Cash on delivery	39 (63.9)	28 (75.7)	9 (34.6)	0 (0.0)
Mobile money	0 (0.0)	0 (0.0)	15 (57.7)	3 (100.0)
Bitcoin or other cryptocurrencies	3 (4.9)	0 (0.0)	5 (19.2)	0 (0.0)
Other^6^	16 (26.2)	10 (27.0)	9 (34.6)	2 (66.7)
Unclear	4 (6.6)	4 (10.8)	1 (3.8)	0 (0.0)

^1^Other countries of domain registrants included Australia, Bahamas, Burundi, Canada, Czech Republic, Great Britain, Iceland, Pakistan, Russia, Spain, and Turks and Caicos Island.

^2^Other countries of website server locations included Canada, France, Germany, Lithuania, Netherlands, Russia, Singapore, and United Arab Emirates.

^3^We checked for discounts on offers pages and the homepage only.

^4^Social media platforms included Facebook, X (formerly Twitter), Instagram, LinkedIn, TikTok, and YouTube.

^5^Additional language options included a mixture of regional (e.g., Hindi, Bangla, etc.) and other national languages (e.g., French, Spanish, etc.).

^6^Other payment methods primarily included Bonga points, Umba loan and gift certificates for Kenya and several “pay later” options in India (Simpl, Zestmoney, etc.).

Just under half of website domains were registered within the respective country (46% for both Kenya and India), with registration in the US also being common (38% for India; 23% for Kenya). The website server was less likely to be in the study country (37% for India; none for Kenya), with a US location being common (36% and 50% for India and Kenya, respectively).

In terms of commercial strategies, a majority of websites serving India (85%) and Kenya (69%) displayed discounts for medicines on the homepage or on a separate ‘offers’ page. Most websites in India (93%) and Kenya (81%) maintained a social media presence, with Facebook and X (formerly Twitter) being the most common. All websites used English, with less than 20% in both Kenya and India having an additional language. While 81% of the websites serving Kenya displayed customer reviews and testimonials, only 44% did so in India. Most websites offered multiple payment options. For India, the most common online payment methods were credit/debit cards, net banking, Unified Payments Interface (UPI) and electronic wallets. In contrast, in Kenya mobile money was the most common payment method, followed by credit/debit cards. Cash on delivery was accepted by 64% of Indian websites and 35% of Kenyan websites.

Comparing websites and apps serving India, apps were slightly more likely than websites to offer other products for sale (OTCs, TCAMs, nutraceuticals), and to offer consultation or lab services, but less likely to provide customer reviews, and very unlikely to deliver outside of India (Table 1). However, when comparing website and app characteristics just for those websites with associated apps, there was little difference in the offer of consultations or other (non-POM) products (S2 Table). It was challenging to make comparisons between websites and apps for Kenya as there were only 3 apps.

### Compliance with best practices in India and Kenya

Table 2 presents findings on compliance to best practice for websites and apps. Overall, 42% of websites serving Kenya provided complete information on tracer medicines (i.e., indications, side-effects, drug interactions, and contraindications), compared with only 16% serving India. However, 90% of sites serving India had a prescription upload facility, compared with only 58% in Kenya. For both countries, prescription upload was most commonly through the website, though over a third of websites serving Kenya offered upload through WhatsApp.

**Table 2:** Compliance with best practices by e-pharmacies: overall, by visit numbers, and by location, n (%).

	Overall				Websites by visit numbers				Websites by physical location^1^			
	India		Kenya		India		Kenya		India		Kenya	
	Websites	Apps	Websites	Apps	Top 20% by visits	Other websites	Top 20% by visits	Other websites	Physical location in India	Other websites	Physical location in Kenya	Other websites
Number of e-pharmacies reviewed (N)	**61**	**37**	**26**	**3**	**13**	**48**	**5**	**21**	**56**	**5**	**19**	**7**
Information provided on medicines^2^												
*Uses*	35 (57.4)	20 (54.0)	12 (46.1)	1 (33.3)	9 (69.2)	26 (54.2)	3 (60.0)	9 (42.9)	30 (53.6)	5 (100.0)	6 (31.6)	6 (85.7)
*Side-effects*	32 (52.5)	20 (54.0)	12 (46.1)	1 (33.3)	9 (69.2)	23 (47.9)	3 (60.0)	9 (42.9)	28 (50.0)	4 (80.0)	6 (31.6)	6 (85.7)
*Drug interactions*	15 (24.6)	9 (24.3)	11 (42.3)	1 (33.3)	8 (61.5)	7 (14.6)	3 (60.0)	8 (38.1)	12 (21.4)	3 (60.0)	6 (31.6)	5 (71.4)
*Contraindications*	15 (24.6)	11 (29.7)	12 (46.1)	1 (33.3)	7 (53.9)	8 (16.7)	3 (60.0)	9 (42.9)	13 (23.2)	2 (40.0)	6 (31.6)	6 (85.7)
*All the above*	10 (16.4)	7 (18.9)	11 (42.3)	1 (33.3)	6 (46.2)	4 (8.3)	3 (60.0)	8 (38.1)	8 (14.3)	2 (40.0)	6 (31.6)	5 (71.4)
Option to upload a prescription												
*On website/app*	49 (80.3)	37 (100.0)	11 (42.3)	1 (33.0)	13 (100.0)	36 (75.0)	2 (40.0)	9 (42.9)	49 (87.5)	0 (0.0)	10 (52.6)	1 (14.3)
*On WhatsApp*	10 (16.4)	4 (10.8)	9 (34.6)	0 (0.0)	2 (15.4)	8 (16.7)	4 (80.0)	5 (23.8)	10 (17.9)	0 (0.0)	8 (42.1)	1 (14.3)
*Via e-mail*	7 (11.5)	1 (2.7)	0 (0.0)	0 (0.0)	1 (7.7)	6 (12.5)	0 (0.0)	0 (0.0)	5 (8.9)	2 (40.0)	0 (0.0)	0 (0.0)
*Others (fax, post, etc.)*	3 (4.9)	0 (0.0)	1 (3.8)	0 (0.0)	0 (0.0)	3 (6.3)	0 (0.0)	1 (4.8)	1 (1.8)	2 (40.0)	1 (5.3)	0 (0.0)
*Unavailable*	6 (9.8)	0 (0.0)	11 (42.3)	2 (66.7)	0 (0.0)	6 (12.5)	0 (0.0)	11 (52.4)	3 (5.4)	3 (60.0)	6 (31.6)	5 (71.4)
Does not display narcotics or controlled substances for sale^3^	40 (65.6)	23 (62.2)	10 (38.5)	1 (33.3)	11 (84.6)	29 (60.4)	2 (40.0)	8 (38.1)	35 (62.5)	5 (100.0)	9 (47.4)	1 (14.3)
Does not display product-specific advertisements for POMs	61 (100.0)	37 (100.0)	25 (96.1)	3 (100.0)	13 (100.0)	48 (100.0)	5 (100.0)	20 (95.2)	56 (100.0)	5 (100.0)	18 (94.7)	7 (100.0)
Provides refill reminders	8 (13.1)	15 (40.5)	1 (4.2)	0 (0.0)	3 (23.1)	5 (10.4)	0 (0.0)	1 (5.3)	8 (14.3)	0 (0.0)	1 (5.6)	0 (0.0)
Provides a complete physical address	49 (80.3)	25 (67.6)	20 (76.9)	3 (100.0)	13 (100.0)	36 (75.0)	4 (80.0)	16 (76.2)	47 (83.9)	2 (40.0)	17 (89.5)	3 (42.9)
Provides a telephonic helpline	57 (93.4)	29 (78.4)	26 (100.0)	3 (100.0)	10 (76.9)	47 (97.9)	5 (100.0)	21 (100.0)	52 (92.9)	5 (100.0)	19 (100.0)	7 (100.0)
Provides assistance via text chat												
*On website/app*	17 (27.9)	12 (32.4)	6 (23.1)	1 (33.3)	4 (30.8)	13 (27.1)	3 (60.0)	3 (14.3)	15 (26.8)	2 (40.0)	5 (26.3)	1 (14.3)
*On WhatsApp*	18 (29.5)	11 (29.7)	17 (65.4)	1 (33.3)	1 (7.7)	17 (35.4)	4 (80.0)	13 (61.9)	18 (32.1)	0 (0.0)	15 (79.0)	2 (28.6)
Provides tracking of delivery	38 (62.3)	18 (48.6)	15 (57.7)	2 (66.7)	10 (76.9)	28 (58.3)	3 (60.0)	12 (57.1)	34 (60.7)	4 (80.0)	11 (57.9)	4 (57.1)
Provides name and details of the pharmacy director/superintendent/owner	17 (27.9)	6 (16.2)	1 (3.9)	0 (0.0)	5 (38.5)	12 (25.0)	1 (20.0)	0 (0.0)	17 (30.4)	0 (0.0)	1 (5.3)	0 (0.0)
Provides registration details of the e-pharmacy	3 (4.9)	0 (0.0)	5 (19.2)	1 (33.3)	1 (7.7)	2 (4.2)	1 (20.0)	4 (19.1)	2 (3.6)	1 (20.0)	5 (26.3)	0 (0.0)
Provides registration details of the pharmacist	0 (0.0)	0 (0.0)	1 (3.9)	0 (0.0)	0 (0.0)	0 (0.0)	1 (20.0)	0 (0.0)	0 (0.0)	0 (0.0)	1 (5.3)	0 (0.0)
Displays customers’ privacy policy	53 (86.9)	31 (83.8)	17 (65.4)	3 (100.0)	13 (100.0)	40 (83.3)	4 (80.0)	13 (61.9)	50 (89.3)	3 (60.0)	14 (73.7)	3 (42.9)
Displays procedure for grievance redressal	12 (19.7)	16 (43.2)	0 (0.0)	0 (0.0)	8 (61.5)	4 (8.3)	0 (0.0)	0 (0.0)	12 (21.4)	0 (0.0)	0 (0.0)	0 (0.0)

^1^Based on physical address, stated location or on phone number. “Other” category includes websites with physical location outside of study country, or where location could not be ascertained.

^2^Information on medicines was assessed for selected ‘tracer medicines’, which include some of the most commonly sold prescription-only medicines in India and Kenya.

^3^The few websites that did not display a browsable product catalogue were coded as not displaying narcotics or controlled substances for sale, though we note that it might have been possible to purchase these after uploading a prescription.

Although it is not considered best practice to sell narcotic or controlled substances online, 62% of websites did so in Kenya, compared with 34% in India. All websites serving India, and all but one serving Kenya, complied with the practice of prohibiting advertisements of POMs.

Providing refill reminders (an automated system that notifies the user to refill their prescriptions every month) was rare. Only 13% of websites serving India did so, and only one in Kenya. All websites serving Kenya and 93% serving India provided a telephonic helpline, but a much smaller proportion of websites provided assistance via text chat, either through WhatsApp (Kenya: 65% and India: 30%) or on their website (Kenya: 23% and India: 28%). Delivery tracking was provided by 62% of websites serving India and 58% serving Kenya.

Only 5% of websites serving India and 19% serving Kenya displayed a registration number for the e-pharmacy. Moreover, barring one e-pharmacy in Kenya, no websites displayed details of the supervising pharmacist(s). Details of the owner, director or supervisory board were provided by only 28% and 4% of websites serving India and Kenya respectively. Most (87%) websites serving India displayed the customers’ privacy policy, whereas 65% of the websites serving Kenya did so. A procedure for grievance redressal was displayed by 20% of websites serving India, but none serving Kenya.

Comparing websites and apps serving India, websites were more likely to provide a physical address, a telephone helpline, delivery tracking, the name of the director/owner/board, and e-pharmacy registration details (Table 2). However, apps were more likely to offer prescription upload, refill reminders and a grievance procedure. Similar results were found when comparing best practices only for websites that had associated apps (S3 Table).

Table 2 also shows results for websites stratified by website traffic and by physical location. The top 20% of websites in terms of visits (high-traffic websites) in India (n = 13) and Kenya (n = 5) were compared with the remaining websites. Similarly, websites with offices/facilities physically located in the study country (determined by listed physical address, stated location or phone number) (n = 56 for India; n = 19 for Kenya) are compared with those located outside these countries or whose physical location could not be ascertained. In both countries, all of the websites in the top 20% by visits were physically based in the study country.

Websites with high-traffic volume were more likely to display complete medicine information, provide a prescription upload facility, and display a privacy policy in both countries. In India, high-traffic websites were also more likely to display a grievance policy, provide delivery tracking, and refrain from selling narcotics or controlled drugs, though there was little or no difference in these outcomes by traffic volume in Kenya. Surprisingly telephone helplines were more common among low-traffic websites in India.

Websites for e-pharmacies based inside the respective study countries were more likely to provide a prescription upload facility, and to display a privacy policy and physical address details in both countries. For India, e-pharmacies located in the country were also more likely to provide refill reminders and display grievance policies and ownership details. For Kenya e-pharmacies located internally were more likely to provide registration details. Further, for Kenya, websites located internally were less likely to sell narcotics or controlled substances, but the opposite was true in India, where websites located internally were more likely to sell these products. In both countries, complete drug information was more likely to be provided by websites outside of the country.

### Compliance with regulatory requirements in Kenya

Table 3 reports compliance of Kenyan websites with the regulatory requirements. None of the 26 e-pharmacies serving Kenya complied with all 12 requirements. On average, websites serving Kenya complied with 8.9 of the 12 (74%) requirements. High-traffic websites complied with a higher number of requirements (10.0) compared to the other websites (8.7). Similarly, websites located in Kenya (9.4) outperformed those located elsewhere (7.7).

**Table 3:** Compliance with regulatory requirements for Kenyan websites, n (%).

	Overall	By visit numbers		By physical location	
**Regulatory requirements** [17]	**N (%)**	**Top 20% by visits**	**Other websites**	**Physical location in Kenya** ^1^	**Other websites**
**Number of e-pharmacies reviewed (N)**	**26**	**5**	**21**	**19**	**7**
Displays a physical address	20 (76.9)	4 (80.0)	16 (76.2)	17 (89.5)	3 (42.9)
Provides a helpline	26 (100.0)	5 (100.0)	21 (100.0)	19 (100.0)	7 (100.0)
Provides a phone number	26 (100.0)	5 (100.0)	21 (100.0)	19 (100.0)	7 (100.0)
Provides an e-mail address	21 (80.8)	5 (100.0)	16 (76.2)	18 (94.7)	3 (42.9)
Displays the health safety code	8 (30.8)	2 (40.0)	6 (28.6)	7 (36.8)	1 (14.3)
Displays EV-SSL certificate^2^	26 (100.0)	5 (100.0)	21 (100.0)	19 (100.0)	7 (100.0)
Displays customers’ privacy policy	17 (65.4)	4 (80.0)	13 (61.9)	14 (73.7)	3 (42.9)
Provides facility to upload prescriptions	15 (57.7)	5 (100.0)	10 (47.6)	13 (68.4)	2 (28.6)
Does not sell narcotic or controlled substances^3^	10 (38.5)	2 (40.0)	8 (38.1)	9 (47.4)	1 (14.3)
Does not engage in illegal advertisements of prescription-only medicines	25 (96.2)	5 (100.0)	20 (95.2)	18 (94.7)	7 (100.0)
Provides information on contraindications and side effects	12 (46.2)	3 (60.0)	9 (42.9)	6 (31.6)	6 (85.7)
Language used is either English or Swahili	26 (100.0)	5 (100.0)	21 (100.0)	19 (100.0)	7 (100.0)
**Complies with all the above**	**0 (0.0)**	**0 (0.0)**	**0 (0.0)**	**0 (0.0)**	**0 (0.0)**
**Mean number of regulations complied with (out of 12)**	**8.9**	**10.0**	**8.7**	**9.4**	**7.7**

^1^Determined based on the physical address displayed, and for those that did not display a physical address, on stated location or on the country code of the phone number provided. “Other websites” category includes websites with physical location outside of study country, or where location could not be ascertained.

^2^EV-SSL: Extended Validation secure sockets layer (the website has completed a 16-point check to verify details such as: website domain, website owner, and the applicant’s legal, physical, and operational existence and identity).

^3^Based on the products displayed for sale on the website. Websites that did not display a browsable product catalogue were coded as not selling narcotics or controlled substances, though it might have been possible to purchase these after uploading a prescription.

See (S2 File) for the 2022 requirements.

Among assessed requirements, the lowest level of compliance (31%) was observed for the display of the health safety code (indicating authorisation by the Pharmacy and Poisons Board), although compliance was higher among higher-traffic websites (40% vs. 29%) and among those physically located in Kenya (37% vs. 14%).

### Compliance with ‘proposed requirements’ in India

Table 4 reports compliance of Indian websites with the ‘proposed requirements’ for India, based on a range of existing regulations, guidelines, and draft instruments. None of the 61 websites met all the ‘proposed requirements’, and on average, websites complied with 7.5 of the 14 requirements. High-traffic websites complied with a higher number of requirements (8.7) compared to other websites (7.1), as did websites located in India (7.6) compared to those based elsewhere (5.6).

**Table 4:** Compliance with proposed requirements for Indian websites, n (%).

	Overall	By visit numbers		By physical location	
	**N (%)**	**Top 20% by visits**	**Other websites**	**Physical location in India** ^1^	**Other websites**
**Number of e-pharmacies (N)**	**61**	**13**	**48**	**56**	**5**
Current regulatory requirements applicable to e-pharmacy					
Displays customers’ privacy policy [22,25]	53 (86.9)	13 (100.0)	40 (83.3)	50 (89.3)	3 (60.0)
Displays a detailed procedure for grievance redressal [23]	12 (19.7)	8 (61.5)	4 (8.3)	12 (21.4)	0 (0.0)
Displays return policy [23]	53 (86.9)	13 (100.0)	40 (83.3)	48 (85.7)	5 (100.0)
Does not display advertisements of prescription-only medicines [26]	61 (100.0)	13 (100.0)	48 (100.0)	56 (100.0)	5 (100.0)
Has a physical address in the country [24]	47 (83.9)	13 (100.0)	34 (79.1)	47 (83.9)	0 (0.0)
Additional requirements from draft bills and existing guidelines					
Displays information on authorisation from the CDSCO [21]	0 (0.0)	0 (0.0)	0 (0.0)	0 (0.0)	0 (0.0)
Displays registration number of the pharmacy [21]	3 (4.9)	1 (7.7)	2 (4.2)	2 (3.6)	1 (20.0)
Provides names and details of the director/ superintendent/ owner [21]	17 (27.9)	5 (38.5)	12 (25.0)	17 (30.4)	0 (0.0)
Displays name and registration details of the pharmacist [21]	0 (0.0)	0 (0.0)	0 (0.0)	0 (0.0)	0 (0.0)
Displays complete contact information [21]					
Phone	57 (93.4)	11 (84.6)	46 (95.8)	52 (92.9)	5 (100.0)
Email address	57 (93.4)	13 (100.0)	44 (91.7)	54 (96.4)	3 (60.0)
Physical address	49 (80.3)	13 (100.0)	36 (75.0)	47 (83.9)	2 (40.0)
Does not sell Schedule X and/or habit-forming substances^2^ [19]	40 (65.6)	11 (84.6)	29 (60.4)	35 (62.5)	5 (100.0)
Does not deliver outside India [19]	34 (55.7)	7 (53.9)	27 (56.3)	34 (60.7)	0 (0.0)
**Complies with all the above**	**0 (0.0)**	**0 (0.0)**	**0 (0.0)**	**0 (0.0)**	**0 (0.0)**
**Mean number of regulations/best practices complied with (out of 14)**	**7.5**	**8.7**	**7.1**	**7.6**	**5.6**

^1^Determined based on the physical address displayed, and for those that did not display a physical address, it was determined based on stated location or on the country code of the phone number provided. The “other” category includes websites with physical location outside of study country, or where location could not be ascertained.

^2^Schedule X drugs are a subset of prescription-only drugs for which the prescription must be preserved by the retailer for two years. These include controlled drugs, such as narcotics, psychotropic, and other habit-forming drugs. This indicator was evaluated based on what products were displayed for sale on the website. Websites without a browsable product catalogue were coded as not selling Schedule X/ habit-forming substance, though it might have been possible to purchase these after uploading a prescription.

CDSCO: Central Drugs Standard Control Organisation.

None of the Indian websites displayed authorisation information from the regulatory authority, the Central Drugs Standard Control Organisation (CDSCO), or the name and registration details of the pharmacist. Compliance was also low for displaying a registration number (5%), and even for those listing a number, it was unclear which regulatory body they claimed to be registered with. Only 20% displayed a detailed procedure for grievance redressal, including the name and contact details of the grievance officer, which is required by Indian e-commerce regulations, though compliance with this requirement was greater among high-traffic websites (62%).

## Discussion

We examined e-pharmacy websites and apps selling POMs to consumers in India and Kenya, two LMICs at contrasting stages of e-pharmacy market and regulatory development. While a few studies have reported on compliance of LMIC e-pharmacies to a limited scope of regulations based on website reviews [28] or mystery shoppers [11,12], we are aware of only one other study that assessed compliance to a comprehensive set of regulations/best practices. [29] To our knowledge, ours is the first study to systematically assess e-pharmacy mobile apps in any income setting.

A number of limitations should be noted. First, we were only able to evaluate compliance with the subset of best practices and regulations that could be assessed through website/app review. While this allows exploration of a number of key practices, it does not permit assessment of sales behaviour in practice, for instance, whether medicines were only sold with a valid prescription, the advice provided to specific clients, or medicine packaging. It also does not allow us to assess compliance with regulations related to e-pharmacy management such as staff qualifications. Secondly, we reported the information drawn from the websites/apps at face value and did not subject it to authenticity or validation checks. For example, we did not check whether registration numbers provided were valid, or whether helplines were functional. Third, we reported the availability of drug information on side-effects, drug interactions and contraindications, but did not assess whether this information was accurate. Finally, we did not assess the additional regulatory requirements added in the April 2023 update of the Kenyan Guidelines, such as displaying policies on counselling patients and delivery of medicines, the hours or service provision, and the PPB contact details; [17] however, all 12 requirements from the 2022 Guidelines that we did assess were also included in the 2023 Guidelines. Given these limitations, the results can be considered as minimum estimates of non-compliance with best practices and regulation.

### Market structure

Both country populations were served by many e-pharmacy businesses: 65 websites and apps in India and 26 in Kenya of which at least 51 were based in India and 19 in Kenya. While there were 2.5 times more e-pharmacies serving India, the population to e-pharmacy ratio was actually much lower in Kenya (2 million in Kenya compared to 21 million in India). [30] Supplemental analysis of e-pharmacy market dynamics one year after the original search found that the number of e-pharmacy websites had increased from 61 to 76 in India and 26 to 28 in Kenya, with new market entrants (19 in India, 10 in Kenya) tending to be physically based in the study country, and market leavers (4 in India, 8 in Kenya) based externally. The number of e-pharmacies may appear quite high, but it is very low compared with an estimated 850,000 licensed brick-and-mortar pharmacies nationwide in India, [31] and over 5,000 in Kenya, [32] most of which remain independently owned. [33] While e-pharmacy markets present regulatory challenges, the relatively small number of providers to control implies that they also present opportunities to enhance pharmacy regulation.

### Compliance to regulations and best practices

Overall, compliance to regulations and guidelines was low across both countries, with e-pharmacies serving Kenya complying (on average) with 8.9 of 12 regulations, and those in India with 7.5 of 14 ‘proposed requirements’. None of the e-pharmacies in either country was fully compliant. The higher overall compliance for Kenya could reflect the existence of explicit e-pharmacy-specific regulations. In India, there was better adherence to requirements based on existing (general) regulations. Most fundamentally, few e-pharmacies provided evidence of registration or authorisation by national regulators: only 31% serving Kenya and none serving India displayed the required regulatory verification (health safety code in Kenya and CDSCO authorisation in India). Similar findings were observed in a study from Bangladesh, where no single e-pharmacy displayed licensure details or permission documents [28]. A retrospective check on the Kenyan regulator’s website revealed that 14 (54%) of the e-pharmacies were associated with registered brick-and-mortar pharmacies in Kenya, as prescribed by guidelines, meaning that more e-pharmacies may be registered than stated on their websites (similar checks were not possible in India). However, the low display of registration codes still raises important concerns, given the common presence of ‘rogue’, unregistered e-pharmacies reported globally [34,35].

Other major areas of concern were the lack of a prescription upload facility in 42% of websites serving Kenya, and 10% serving India, implying that POMs were very likely sold without a prescription; and the offer of narcotics or controlled substances for sale in 62% of websites serving Kenya (despite regulatory prohibition against their online sale) and 34% serving India. Previous studies in LMICs have highlighted similar concerns for various classes of POMs, including antibiotics, antihypertensives and abortifacients [11,12,36]. These practices raise important risks for patient safety and public health. Selling POMs without the authorisation of a physician can have harmful consequences when medicines are used incorrectly. In the case of antibiotics, resulting overuse may lead to the selection and spread of antimicrobial resistance [37,38]. The anonymity of internet transactions may facilitate the purchase of narcotics and other controlled substances, such as opioids, with sale of narcotics online without a prescription already widely reported in HIC settings [39]. This could also be an important risk in LMICs, where non-medicinal use of controlled substances has been widely reported [40,41], which can result in devastating effects, including addiction and overdose. However, it should be noted that these practices are not specific to online pharmacies. There is substantial evidence of high prevalence of sales of POMs without prescription in brick-and-mortar pharmacies across most LMICs [42,43], including narcotics/controlled drugs [40,43]. Going forward it is possible that the more consolidated nature of the e-pharmacy market, combined with the traceability of online transactions, could facilitate more effective audit of prescription medicine dispensing.

Other common areas of non-compliance included failure to display contact information, and policies on grievance redressal or privacy. A particular concern was the lack of information displayed on medicine side-effects, drug interactions, indications, and contraindications. Better information provision was reported in a review of 100 e-pharmacy websites across 17 countries (including India), which found only 27% failed to provide information on side-effects, and 38% on contraindications [29], though 12 of the countries covered were high-income, and therefore would be expected to have stronger regulatory systems. Provision of drug-related information online is a “low-hanging fruit”, which would be relatively easy for e-pharmacies to address and for regulators to monitor.

### Implications for regulatory policy

A basic prerequisite for good governance of the e-pharmacy market is the presence of a clear set of e-pharmacy specific regulations. Kenya has made important strides in this area and is an early mover compared to other African countries [5]. By contrast in India, several draft rules have been pending approval since 2018, with delays reflecting both safety concerns about online sales, and a fear among brick-and-mortar pharmacies that e-pharmacies will drive them out of business. This has resulted in nationwide strikes calling for the ban of online pharmacies, as well as a series of court cases [7,44].

Our research also highlighted the importance of ensuring that regulatory scope covers all aspects of e-pharmacy operations, rather than simply the sale of drugs from individual websites. First, our data documents the growing importance of e-pharmacy apps, particularly in India, with general e-commerce trends in LMICs suggesting that the share of online purchases via apps rather than through websites, will continue to grow rapidly. However, barring a requirement to display the links for the apps’ programming interface in Kenya, apps were not specifically mentioned in e-pharmacy regulations or guidelines from either country. Secondly, a significant minority of e-pharmacies also provided teleconsultations with physicians and/or laboratory services, with demand for such integrated ‘one-stop’ care also expected to grow. These additional services require coordination between multiple regulatory stakeholders—beyond those monitoring pharmacy practice only—as the boundaries between these business models increasingly become blurred. However, there was no mention of telemedicine in the Kenyan regulations or Indian draft rules or Code of Conduct. Finally, the role of aggregators and marketplaces is growing in the e-pharmacy space, also leading to regulatory complications and opportunities. While there are concerns that they may avoid liability for the actions of the organisations they advertise, there is also potential for marketplaces to play a role in “decentred regulation” by restricting participation on their platforms to e-pharmacies which adhere to government rules [45].

While overall compliance with regulations/guidelines was low, there was considerable variation, with better compliance among the higher-traffic websites and those located within the study countries. This raises questions about how resources should be prioritised in tackling non-compliant practices. Recent thinking in regulatory policy highlights the potential benefits of risk-based regulation, which involves prioritising resources to regulatees expected to be highest risk [46], an approach that is starting to be adopted in LMIC settings [47,48]. However, while higher-traffic providers had better compliance, regulators may still want to focus their efforts on these providers for the very reason that they serve a higher number of clients. In addition, these largely compliant e-pharmacies may be more amenable to engaging with regulators and cooperating in the development and implementation of regulation. Overall, regulators may want to consider a tailored, risk-based regulatory approach, where they work with the largely compliant e-pharmacies (“the good”) to enhance regulatory and accreditation systems; improve enforcement among the partially compliant (“the bad”) and eliminate the largely non-compliant from the market altogether (“the ugly”). As a rough categorisation, our analysis indicates that the top 20% of e-pharmacies by visits might correspond to the “good” category, with those physically based outside of the study country or for which location was unclear serving as examples of the “ugly”. Previous studies have highlighted various rogue practices used by online pharmacies selling internationally, including selling medicines without a prescription and providing inappropriate discounts and bonuses [10]. In this study we encountered similar practices among websites located outside the study country, such as offering “bonus sildenafil pills” (for erectile dysfunction) with every order, or promoting “party pills” and “performance enhancers”. However, while e-pharmacies based outside of the study country had poorer compliance, national regulators also have limited scope to impose regulations on firms outside their jurisdiction and operating across international borders. Options that could be considered include blocking specific websites or cooperating with search engines to deprioritise them, though rogue e-pharmacies are known to regularly change their web addresses and domain names in response to such actions [35]. Ultimately, international cooperation is likely to be required to control the worst offenders operating across multiple countries.

Priorities for future research on LMIC e-pharmacies include investigating sales practices by recording the details of real clients or mystery shoppers [11,36,49], and exploring the integration of telemedicine and e-pharmacy services, and the growing sale of medicines on social media platforms. Going forward, a key priority will be to test and evaluate regulatory innovations and their implications for both regulatory compliance and improving equity of access to medicines.

## Conclusion

The compliance of e-pharmacies with regulations in Kenya and ‘proposed requirements’ in India was low. However, there was considerable variation, with better compliance to mandatory regulations, and by the most visited e-pharmacies and those based within the study countries. These findings can be leveraged to direct more targeted and efficient regulatory enforcement in Kenya, and to inform the development of a comprehensive regulatory framework for e-pharmacy in India. We recommend that risk-based regulatory approaches should be tailored such that regulators work with the largely compliant (“good”) e-pharmacies, improve enforcement among the partially compliant (“bad”), and eliminate the largely non-compliant (“ugly”) e-pharmacies.

## Supporting information

S1 Table Regulatory requirements in India and Kenya. (DOCX)

S1 File Website review tool, including the data dictionary and codebook. (PDF)

S2 Table E-pharmacy characteristics and business practices for websites with associated apps only. (DOCX)

S3 Table Compliance with best practices for websites with associated apps only. (DOCX)

S2 File Republic of Kenya: Ministry of Health, Pharmacy and Poisons Board, Guidelines for Internet Pharmacy Services in Kenya (2022). (PDF)

## References

[1] World Health Organization. Ten years in public health, 2007-2017—Report by Dr Margaret Chan, Director-General—Access to Medicines: making market forces serve the poor [Internet]. Geneva, Switzerland: World Health Organization; 2017 [cited 2024 Oct 9]. Available from: https://iris.who.int/bitstream/handle/10665/255355/9789241512442-eng.pdf

[2] Alliance for Safe Online Pharmacy. Unlocking the benefits of online access to prescription medicines across the EU [Internet]. Copenhagen, Denmark: Copenhagen Economics; 2024 Jan 30 [cited 2024 Oct 9]. Available from: https://copenhageneconomics.com/wp-content/uploads/2024/02/Copenhagen-Economics-Unlocking-the-benefits-of-online-access-to-prescription-medicines_2024.pdf

[3] Future Market Insights. Newark (DE), US: Future Market Insights Inc. ePharmacy Market by Product & Region - Forecast 2023 to 2033 [Internet]; 2023 Feb [cited 2024 Oct 9]. Available from: https://www.futuremarketinsights.com/reports/epharmacy-market

[4] IBEF. New Delhi, India: Indian Brand Equity Foundation. E-pharmacies and Online Pharma Distribution Companies in India; 2023 Jul [cited 2024 Oct 9] Available from: https://www.ibef.org/blogs/e-pharmacies-and-online-pharma-distribution-companies-in-india.E-pharmacies and online pharma distribution companies in India. Indian Brand Equity Foundation. 2023.

[5] Afuye S, Uka J, Lawal D, Adeseun R, Staples MH. Advancing access to essential health products via online pharmacies in Africa: Regulatory Landscaping Report [Internet]. Vancouver (BC), Canada: Salient Advisory. 2023 Sep [cited 2024 Oct 9]. Available from: https://www.salientadvisory.com/reports/report-online-pharmacy-in-africa-regulatory-landscape-opportunities-for-action/

[6] Miller R, Wafula F, Onoka CA, Saligram P, Musiega A, Ogira D, et al. When technology precedes regulation: the challenges and opportunities of e-pharmacy in low-income and middle-income countries. BMJ Glob Health. 2021;6(5):e005405. doi: 10.1136/bmjgh-2021-005405 34016578 PMC8141442

[7] Satheesh G, Puthean S, Chaudhary V. E-pharmacies in India: can they improve the pharmaceutical service delivery? J Glob Health. 2019;10:010301. 32082543 10.7189/jogh.10.010302 PMC7007514

[8] LegitScript LLC. The Internet Pharmacy Market in 2016: Trends, Challenges, and Opportunities [Internet]. Washington, DC, USA: Center for Safe Internet Pharmacies; 2016 Jan [cited 2024 Oct 9]. Available from: http://safemedsonline.org/wp-content/uploads/2016/01/The-Internet-Pharmacy-Market-in-2016.pdf

[9] Limbu Y, Huhmann B. Illicit online pharmacies: a scoping review. Int J Environ Res Public Health. 2023;20(9):5748. 37174265 10.3390/ijerph20095748 PMC10178756

[10] Long C, Kumaran H, Goh K, Bakrin F, Ming L, Rehman I, et al. Online pharmacies selling prescription drugs: systematic review. Pharmacy. 2022;10(2):42. 35448701 10.3390/pharmacy10020042 PMC9031186

[11] Gong Y, Jiang N, Chen Z, Wang J, Zhang J, Feng J, et al. Over-the-counter antibiotic sales in community and online pharmacies, China.. Bull World Health Org. 2020;98(7):449–57. 32742030 10.2471/BLT.19.242370 PMC7375218

[12] Jiang N, Yin X, Chen Z, Li H, Wang J, Zhang J, et al. The sale of antihypertensive drugs by online pharmacies in China: A nationwide cross-sectional survey. Eur J Prev Cardiol. 2020;27(19):2334–7. doi: 10.1177/2047487319896678 31914793

[13] Hall A, Antonopoulos GA. License to pill: illegal entrepreneurs’ tactics in the online trade of medicines. In: van Duyne PC, Maljevic A, Antonopoulos GA, Harvey J, von Lampe K, editors. The relativity of wrongdoing: corruption, organised crime, fraud and money laundering in perspective. Nijmegen: Wolf Legal Publisher; 2015:229–52.

[14] Harris PA, Taylor R, Thielke R, Payne J, Gonzalez N, Conde JG, et al. Research electronic data capture (REDCap)–a metadata-driven methodology and workflow process for providing translational research informatics support. J Biomed Inform. 2009;42(2):377–81. doi: 10.1016/j.jbi.2008.08.010 18929686 PMC2700030

[15] Hock SC, Lee MMX, Chan LW. Regulating online pharmacies and medicinal product e-commerce [Internet]. Bethesda(MA), USA: International Society for Pharmaceutical Engineering Inc.; 2019 Nov-Dec [cited 2024 Oct 9]. Available from: https://www.ispe.gr.jp/ISPE/02_katsudou/pdf/202104_en.pdf

[16] National Association of Boards of Pharmacy. Digital pharmacy [Internet]. Mount Prospect: NABP; [cited 2024 Oct 9]. Available from: https://nabp.pharmacy/programs/accreditations/digital-pharmacy

[17] Republic of Kenya: Ministry of health, pharmacy and poisons board. Guidelines for internet pharmacy services in Kenya [Internet]. Nairobi: Ministry of Health, Pharmacy and Poisons Board; 2023 Apr. [cited 2024 Mar 4]. Available from: https://web.pharmacyboardkenya.org/download/guidelines-on-internet-pharmacy-services/?wpdmdl=4446&refresh=67808296db44d1736475286&ind=1696852606205&filename=GUIDELINES-FOR-INTERNET-PHARMACY-SERVICES-IN-KENYA.pdf

[18] Republic of Kenya: Ministry of Health, Pharmacy and Poisons Board. Guidelines for Registration and Licensing of Premises [Internet]. Nairobi: Ministry of Health, Pharmacy and Poisons Board; 2023 Apr. [cited 2024 Mar 4] Available from: https://web.pharmacyboardkenya.org/download/guidelines-for-registration-and-licensing-of-premises/?wpdmdl=4423&refresh=67808388204391736475528&ind=1696853155842&filename=GUIDELINES-FOR-REGISTRATION-AND-LICENSING-OF-PREMISES.pdf

[19] Federation of Indian Chambers of Commerce & Industry (FICCI). Self-Regulation Code of Conduct for the e-pharmacy sector in the interest of consumer [Internet]. New Delhi: Federation of Indian Chambers of Commerce & Industry; 2016 Nov 21 [cited 2024 Oct 9]. Available from: https://ficci.in/public/storage/PressRelease/2600/ficci-press-nov21-e-pharmacy.pdf

[20] Government of India: Ministry of Health and Family Welfare. Draft Drugs, Cosmetics, and Medical Devices Bill [Internet]. New Delhi: Ministry of Health and Family Welfare. 2022 Sept [cited 2024 Oct 9]. Available from: https://main.mohfw.gov.in/?q=newshighlights-97

[21] Government of India: Ministry of Health and Family Welfare. Drugs and Cosmetics (Draft) Amendment Rules [Internet]. New Delhi: Ministry of Health and Family Welfare. 2018 Aug 28 [cited 2024 Oct 9]. Available from: https://cdsco.gov.in/opencms/resources/UploadCDSCOWeb/2018/UploadGazette_NotificationsFiles/2018.08.28_Draft%20GSR%20817(E)_Sale%20of%20Drugs%20by%20E-Pharmacy.pdf

[22] Government of India: Ministry of communication and information technology. the information technology (Reasonable Security Practices and Procedures And Sensitive Personal Data Or Information) Rules [Internet]. New Delhi: Ministry of Communication And Information Technology. 2011 Apr 11 [cited 2024 Oct 9]. Available from: https://upload.indiacode.nic.in/showfile?actid=AC_CEN_45_76_00001_200021_1517807324077&type=rule&filename=GSR313E_10511(1)_0.pdf

[23] Government of India: Ministry of consumer affairs, food and public distribution. consumer protection (E-Commerce) rules [Internet]. New Delhi: Ministry of Consumer Affairs, Food and Public Distribution. 2020 Jul 23 [cited 2024 Oct 9]. Available from: https://consumeraffairs.nic.in/theconsumerprotection/consumer-protection-e-commerce-rules-2020

[24] Government of India: Ministry of health and family welfare. Drugs and Cosmetics Act (1940) and Rules (1945) [Internet]. New Delhi: Ministry of Health and Family Welfare. 2016 Dec 31 [cited 2024 Oct 9]. Available from: https://cdsco.gov.in/opencms/export/sites/CDSCO_WEB/Pdf-documents/acts_rules/2016DrugsandCosmeticsAct1940Rules1945.pdf

[25] Government of India: Ministry of information technology. section 43A of the information technology Act (2000) as amended by the information technology (Amendment) Act (IT Act and IT Amendment Act; 2008) [Internet]. New Delhi: Ministry of Information Technology. 2009 Aug [cited 2024 Oct 9]. Available from: https://www.meity.gov.in/content/rules-information-technology-act-2000

[26] Government of India. Drugs and Magic Remedies Act [Internet]. New Delhi: Government of India. 1954 Apr 30 [cited 2024 Oct 9]. Available from: https://www.indiacode.nic.in/bitstream/123456789/1412/1/195421.pdf

[27] StataCorp. Stata statistical software: release 18. College Station, TX: StataCorp LLC. 2023.

[28] Sarker SK, Bhas G, Paul R, Pal H, Yusuf MA, Eva EO, et al. E-Pharmacy utilization on the spectrum of digital pharmaceutical practices, patterns and challenges in Bangladesh. J Shaheed Suhrawardy Med Coll. 2019;11(1):43–7. doi: 10.3329/jssmc.v11i1.43178

[29] Parikh CD, Desai CK, Kiritkumar Shah M, Mishra VR. An evaluation of online pharmacies for compliance to regulatory criteria and price variation of listed medicines. J Young Pharm. 2019;11(2):207–12. doi: 10.5530/jyp.2019.11.43

[30] World Bank, World Development Indicators. Population, total [Internet]. 2024 [cited 2025 Oct 9]. Available from: https://data.worldbank.org/indicator/SP.POP.TOTL

[31] Mugo PM, Mumbi A, Munene D, Nzinga J, Molyneux S, Barasa E, et al. Experiences of and response to the COVID-19 pandemic at private retail pharmacies in Kenya: a mixed-methods study. BMJ Open. 2022;12(6):e058688. doi: 10.1136/bmjopen-2021-058688 35768121 PMC9240447

[32] Miller R, Goodman C. Do chain pharmacies perform better than independent pharmacies? Evidence from a standardised patient study of the management of childhood diarrhoea and suspected tuberculosis in urban India. BMJ Glob Health. 2017;2(3):e000457. doi: 10.1136/bmjgh-2017-000457 29018588 PMC5623271

[33] Wafula F, Onoka C, Musiega A, Okpani A, Ogira D, Ejughemre U, et al. Healthcare clinic and pharmacy chains in Kenya and Nigeria: a qualitative exploration of the opportunities and risks they present for healthcare regulatory systems. Int J Health Plann Manage. 2022;37(6):3329–43. doi: 10.1002/hpm.3560 35983649

[34] Gutorova NO, Pashkov VM, Soloviov OS. Illegal internet pharmacies as a threat to public health in Europe. Wiad Lek. 2021;74(9 cz 1):2169–74. doi: 10.36740/wlek202109125 34725295

[35] LegitScript [Internet]. Portland (OR), USA: LegitScript LLC. Rogue Internet Pharmacies Persist Through Pandemic; 2021 May 4 [cited 2024 Oct 9]. Available from: https://www.legitscript.com/2021/05/04/rogue-internet-pharmacies-persist/

[36] Moore AM, Philbin J, Ariawan I, Budiharsana M, Murro R, Aryanty RI, et al. Online abortion drug sales in Indonesia: a quality of care assessment. Stud Fam Plann. 2020;51(4):295–308. 33079416 10.1111/sifp.12138 PMC7821208

[37] Boyd SE, Moore LSP, Gilchrist M, Costelloe C, Castro-Sánchez E, Franklin BD, et al. Obtaining antibiotics online from within the UK: a cross-sectional study. J Antimicrob Chemother. 2017;72(5):1521–8. doi: 10.1093/jac/dkx003 28333179 PMC5890662

[38] Review on Antimicrobial Resistance. Safe, Secure and Controlled: Managing the supply chain of antimicrobials. [Internet]. London: The Review on Antimicrobial Resistance. 2015 Nov [cited 2024 Oct 9]. Available from: http://amr-review.org/sites/default/files/SafeSecureandControlledShortPaper.pdf

[39] Monteith S, Glenn T. Searching online to buy commonly prescribed psychiatric drugs. Psychiatry Res. 2018;260:248–54. doi: 10.1016/j.psychres.2017.11.037 29220682

[40] Iqbal A, Knaggs R, Anderson C, Toh LS. Logic model for opioid safety in chronic non-malignant pain management, an in-depth qualitative study. Int J Clin Pharm. 2023;45(1):220–32. doi: 10.1007/s11096-022-01493-6 36434367 PMC9702900

[41] Kamba PF, Mulangwa J, Kaggwa B, Kitutu FE, Sewankambo NK, Katabira ET, et al. Compliance of private pharmacies in Uganda with controlled prescription drugs regulations: a mixed-methods study. Subst Abuse Treat Prev Policy. 2020;15(1):16. doi: 10.1186/s13011-020-00261-x 32070374 PMC7027211

[42] Batista AD, A Rodrigues D, Figueiras A, Zapata-Cachafeiro M, Roque F, Herdeiro MT, et al. Antibiotic dispensation without a prescription worldwide: a systematic review. Antibiotics (Basel). 2020;9(11):786. doi: 10.3390/antibiotics9110786 33171743 PMC7694985

[43] Miller R, Goodman C. Performance of retail pharmacies in low- and middle-income Asian settings: a systematic review. Health Policy Plan. 2016;31(7):940–53. doi: 10.1093/heapol/czw007 26962123 PMC4977427

[44] Dcruz AC, Mokashi VN, Pai SR, Sreedhar D. The rise of E-pharmacy in India: Benefits, challenges, and the road ahead. Indian J Pharmacol. 2022;54(4):282–91. doi: 10.4103/ijp.ijp_445_21 36204812 PMC9804119

[45] Hunter BM, Murray SF, Marathe S, Chakravarthi I. Decentred regulation: the case of private healthcare in India. World Dev. 2022;155:105889. doi: 10.1016/j.worlddev.2022.105889 36846632 PMC9941715

[46] Black J. The emergence of risk-based regulation and the new public management in the United Kingdom. In: Masterman R, McHarg A, editors. Public Law. Mytholmroyd: Sweet & Maxwell; 2005. p. 512-–49.

[47] Badr E, Bealchew R, Benton D, Carzaniga A, Das J, Dexter M, et al. World health organization guidance on health practitioner regulation: an overview. J Med Regul. 2024;110(3):5–8.

[48] Republic of Kenya: Ministry of health. quality of care certification manual for the Kenyan health sector 2020. Nairobi: Republic of Kenya Ministry of Health; 2020 Mar [cited 2024 Dec 3]. Available from: https://medbox.org/pdf/6210c2341085b15dcd3d4562

[49] Björnsdottir I, Granas AG, Bradley A, Norris P. A systematic review of the use of simulated patient methodology in pharmacy practice research from 2006 to 2016. Int J Pharm Pract. 2020;28(1):13–25. doi: 10.1111/ijpp.12570 31397533

