# Supplementary material for: The good, the bad, and the ugly: Compliance of e-pharmacies serving India and Kenya with regulatory requirements and best practices
Source: PLOS Glob Public Health. 2025 Feb 3;5(2):e0004202. doi: 10.1371/journal.pgph.0004202 (PMC11790122; doi:10.1371/journal.pgph.0004202)
Supplement: S1 Table — (DOCX) [file pgph.0004202.s001.docx]

**SUPPLEMENTARY FILE**

**S1 Table:** Regulatory requirements in India and Kenya

| **INDIA** | |
| --- | --- |
| ***Current regulatory requirements applicable to e-pharmacy*** | |
| Displays customers’ privacy policy | Section 43A [Information Technology Act (2000) as amended by the Information Technology (Amendment) Act (IT Act and IT Amendment Act; 2008) and Information Technology (Reasonable Security Practices and Procedures and Sensitive Personal Data or Information) Rules (Privacy Rules; 2011)](https://upload.indiacode.nic.in/showfile?actid=AC_CEN_45_76_00001_200021_1517807324077&type=rule&filename=GSR313E_10511(1)_0.pdf) |
| Displays a detailed procedure for grievance redressal | Section 4.2, 4.4 [Consumer Protection (E-Commerce) Rules (2020)](https://consumeraffairs.nic.in/theconsumerprotection/consumer-protection-e-commerce-rules-2020) |
| Displays return policy | Section 7.1(a) [Consumer Protection (E-Commerce) Rules (2020)](https://consumeraffairs.nic.in/theconsumerprotection/consumer-protection-e-commerce-rules-2020) |
| Does not display advertisements of prescription-only medicines | Section 3(d) [Drugs and Magic Remedies Act (1954)](https://www.indiacode.nic.in/bitstream/123456789/1412/1/195421.pdf) |
| Has a physical address in the country | Part VI, Sections 59, 61, 62  [Drugs and Cosmetics Act (1940) and Rules (1945)](https://cdsco.gov.in/opencms/export/sites/CDSCO_WEB/Pdf-documents/acts_rules/2016DrugsandCosmeticsAct1940Rules1945.pdf) |
| ***Additional requirements from draft bills and existing guidelines*** | |
| Displays information on authorization from the CDSCO | Part VIB 67M (3) [Drugs and Cosmetics (Draft) Amendment Rules (2018)](https://cdsco.gov.in/opencms/resources/UploadCDSCOWeb/2018/UploadGazette_NotificationsFiles/2018.08.28_Draft%20GSR%20817(E)_Sale%20of%20Drugs%20by%20E-Pharmacy.pdf) |
| Displays registration number of the pharmacy | Part VIB 67M (3) [Drugs and Cosmetics (Draft) Amendment Rules (2018)](https://cdsco.gov.in/opencms/resources/UploadCDSCOWeb/2018/UploadGazette_NotificationsFiles/2018.08.28_Draft%20GSR%20817(E)_Sale%20of%20Drugs%20by%20E-Pharmacy.pdf) |
| Provides names and details of the director/superintendent/ owner | Part VIB 67M (6) [Drugs and Cosmetics (Draft) Amendment Rules (2018)](https://cdsco.gov.in/opencms/resources/UploadCDSCOWeb/2018/UploadGazette_NotificationsFiles/2018.08.28_Draft%20GSR%20817(E)_Sale%20of%20Drugs%20by%20E-Pharmacy.pdf) |
| Displays name and registration details of the pharmacist | Part VIB 67M (6) [Drugs and Cosmetics (Draft) Amendment Rules (2018)](https://cdsco.gov.in/opencms/resources/UploadCDSCOWeb/2018/UploadGazette_NotificationsFiles/2018.08.28_Draft%20GSR%20817(E)_Sale%20of%20Drugs%20by%20E-Pharmacy.pdf) |
| Displays complete contact information | Part VIB 67M (6) [Drugs and Cosmetics (Draft) Amendment Rules (2018)](https://cdsco.gov.in/opencms/resources/UploadCDSCOWeb/2018/UploadGazette_NotificationsFiles/2018.08.28_Draft%20GSR%20817(E)_Sale%20of%20Drugs%20by%20E-Pharmacy.pdf) |
| Does not sell Schedule X and/or habit-forming substances | Section 2 [India’s Federation of Indian Chambers of Commerce & Industry (FICCI) Self-regulation Code of Conduct (2016)](https://ficci.in/public/storage/PressRelease/2600/ficci-press-nov21-e-pharmacy.pdf) |
| Does not deliver outside India | Section 3 [India’s Federation of Indian Chambers of Commerce & Industry (FICCI) Self-regulation Code of Conduct (2016)](https://ficci.in/public/storage/PressRelease/2600/ficci-press-nov21-e-pharmacy.pdf) |
| **KENYA** | |
| Displays a physical address | Sections 3.4.3 and 10.2.2 |
| Provides a helpline | Sections 3.4.3 and 10.2.2 |
| Provides a phone number | Sections 3.4.3 and 10.2.2 |
| Provides an e-mail address | Sections 3.4.3 and 10.2.2 |
| Displays the health safety code | Sections 3.4.3 and 10.2.1 |
| Displays EV-SSL certificate^2^ | Section 10.2.5 |
| Displays customers’ privacy policy | Sections 3.5 and 3.6 |
| Provides facility to upload prescriptions | Section 12.1(d) |
| Does not sell narcotic or controlled substances | Section 3.15 |
| Does not engage in illegal advertisements of prescription-only medicines | Section 2.6.4 |
| Provides information on contraindications and side effects | Section 6.1.3 |
| Language used is either English or Swahili | Section 6.1.6 |
| *Republic of Kenya: Ministry of Health, Pharmacy and Poisons Board, Guidelines for Internet Pharmacy Services in Kenya (2022) can be found on Supporting Information File 3 | |
