# Supplementary material for: The good, the bad, and the ugly: Compliance of e-pharmacies serving India and Kenya with regulatory requirements and best practices
Source: PLOS Glob Public Health. 2025 Feb 3;5(2):e0004202. doi: 10.1371/journal.pgph.0004202 (PMC11790122; doi:10.1371/journal.pgph.0004202)
Supplement: S1 File — (PDF) [file pgph.0004202.s002.pdf]

Data Dictionary Codebook

23-06-2023 10:09am

#	Variable / Field Name	Field Label<br>*Field Note*	Field Attributes (Field Type, Validation, Choices, Calculations, etc.)
**Instrument: E Pharmacy Website (e_pharmacy_website)**			
1	[record_id]	Record ID	text
2	[e_pharm_name]	Section Header: *Basic Characteristics*<br>1. Name of the e-pharmacy (as mentioned on the site in lowercase without ".com")	text, Required, Identifier
3	[web_add]	2. Complete web address (E.g.: https://www.netmeds.com/ or https://mydawa.com/)	text, Required, Identifier
4	[constitution]	3. Details of the e-pharmacy constitution (Information on directors/supervisors/chairmen/boards etc.)	yesno<br>1 Yes<br>0 No
5	[tel_number]	4. Telephone number of the e-pharmacy Capture the office number. "0" if not provided.	text (number), Required, Identifier
6	[email_add]	5. Email address of the e-pharmacy "np" if not provided.	text, Required, Identifier
7	[phy_add]	6. Is there any physical address for the e-pharmacy?	yesno, Required<br>1 Yes<br>0 No
8	[sec_address]	7. Is there a second address?	yesno<br>1 Yes<br>0 No
9	[state]	8. State (Capture the state wherein the e-pharmacy's physical address is located in lowercase; separate multiple states with a comma without spaces. "np" if not provided.)	text, Required
10	[country]	9. Country If more than one country, capture all, separated by a comma without space.	text, Required
11	[authorization_info]	Section Header: *Authorization details*<br>10. Does the e-pharmacy provide information on authorization from the official licensing body (CDSCO for India)? (refer to "about us" or "FAQ" pages of the e-pharmacy)	radio, Required<br>1 Yes, the site states that they are registered<br>2 Yes, the site states that they are registered and specifies the authorizing body<br>3 No
12	[reg_no]	11. Registration number or the health safety code of the e-pharmacy(E.g.: India: 3184/MIII/20, 3188/MIII/21, 5509/MIII/20B) "np" if not provided.	text, Required, Identifier
13	[pharmacist_details]	12. Does the e-pharmacy display the name and registration details of the pharmacist(s) involved? (refer to "about us" or "FAQ" pages of the e-pharmacy)	yesno, Required<br>1 Yes<br>0 No
14	[e_pharm_app]	Section Header: *Business model*<br>13. Does the e-pharmacy have an app?	yesno, Required<br>1 Yes<br>0 No
15	[track_dlvry]	Section Header: *Customer Service*<br>14. Does the e-pharmacy website state that they provide any tracking of delivery? (Check FAQ or customer support sections)	yesno, Required<br>1 Yes<br>0 No
16	[lang_opt]	15. Are other language options present?	yesno, Required<br>1 Yes<br>0 No
17	[other_lang]<br>Show the field ONLY if:<br>[lang_opt] = '1'	If yes, please specify (Separate multiple languages with a comma without space)	text

18	[tele_helpline]	16. Is a telephonic helpline provided?	radio, Required<br>1 Yes, toll free<br>2 Yes, unspecified

			3 Yes, paid<br>4 No
19	[chat]	17. Is chat option (live or bot) available for customers? Check homepage or "help" sections.	checkbox, Required<br>1 chat___1 Yes, on the website<br>2 chat___2 Yes on Whatsapp<br>3 chat___3 No
20	[faq]	18. Does the e-pharmacy provide an FAQ section? (Check "FAQ" or "Help")	yesno, Required<br>1 Yes<br>0 No
21	[complaints]	19. Does the e-pharmacy display the procedure for complaints in detail? (check FAQ, "complaints", "terms and conditions/policies"; or "contact us" pages)	yesno, Required<br>1 Yes<br>0 No
22	[complaint_url]<br>Show the field ONLY if:<br>[complaints] = '1'	If yes, please provide the URL.	text
23	[review_testimonials]	20. Are customer reviews or testimonials visible on the e-pharmacy website?	yesno, Required<br>1 Yes<br>0 No
24	[return_policy]	21. Does the e-pharmacy display the return policy? (check FAQ, "refunds", or "contact us" pages)	yesno, Required<br>1 Yes<br>0 No
25	[return_url]<br>Show the field ONLY if:<br>[return_policy] = '1'	If yes, please provide the URL.	text
26	[privacy_policy]	Section Header: *Privacy*<br>22. Does the e-pharmacy display customers' privacy policy?	yesno, Required<br>1 Yes<br>0 No
27	[privacy_url]<br>Show the field ONLY if:<br>[privacy_policy] = '1'	If yes, please provide the URL.	text
28	[secur_encrypt]	23. Does the e-pharmacy display any certificates of security/encryption? (E.g., Kenya: Extended Validation SSL (EV-SSL) certificate)	yesno<br>1 Yes<br>0 No
29	[encrypt_certif]<br>Show the field ONLY if:<br>[secur_encrypt] = '1'	If yes, please specify the certificate	text
30	[pay_gateway]	Section Header: *Payment*<br>24. Is a secure payment gateway present? (Redirection to a separate page for payment)	radio, Required<br>1 Yes<br>0 No<br>2 Unclear
31	[pay_options]	25. Available payment options (choose all that apply)	checkbox, Required<br>1 pay_options___1 Credit Card<br>2 pay_options___2 Debit Card<br>3 pay_options___3 Net Banking<br>4 pay_options___4 Store Credit/Wallet<br>5 pay_options___5 Cash on delivery<br>6 pay_options___6 UPI<br>7 pay_options___7 Electronic wallet<br>8 pay_options___8 mPesa/Mobile Money<br>9 pay_options___9 PayPal<br>10 pay_options___10 Health insurance<br>11 pay_options___11 Others

32	[electronic_specify]<br>Show the field ONLY if:<br>[pay_options(7)] = '1'	If electronic wallet, please specify. ("np" if not specified)	text
33	[other_pay]<br>Show the field ONLY if:<br>[pay_options(11)] = '1'	If others, specify the payment method	text
34	[where_deliver]	Section Header: *Coverage*<br>26. Where all does the e-pharmacy deliver to?	radio, Required<br>1 Less than 50% of Indian states<br>2 More than 50% of Indian states<br>3 All over India<br>4 India and Neighbours<br>5 Global<br>6 Unclear<br>7 Other (please specify)
35	[other_geo_cover]<br>Show the field ONLY if:<br>[where_deliver] = '7'	If other, please specify the coverage	text
36	[otc_sale]	Section Header: *Pharmaceutical Aspects*<br>27. Are OTC medicines available for sale? Look for various OTC medicines, if even one is available FOR SALE, go with "Yes" E.g., Paracetamol, pantoprazole, diclofenac, cetirizine, clotrimazole	yesno, Required<br>1 Yes<br>0 No
37	[pom_sale]	28. Are prescription-only medicines available for sale? Look for various POMs, if even one POM is available FOR SALE, go with "Yes" e.g., Amlodipine, insulin, cefixime, atenolol, pregabalin, phenytoin	yesno, Required<br>1 Yes<br>0 No
38	[controlled_sale]	29. Are narcotic or controlled substances available for sale? Look for various narcotic or controlled drugs, if even one is available FOR SALE, go with "Yes" E.g., Tramadol, alprazolam, lorazepam, zolpidem, fentanyl	yesno, Required<br>1 Yes<br>0 No
39	[herbal_altmed]	30. Does the e-pharmacy sell herbal/ alternative medicines? (Check whether "herbal products" or specific products such as arsenicum album, tulsi, aswagandha, or sarpagandha are available for sale)	yesno, Required<br>1 Yes<br>0 No
40	[nutra]	31. Does the e-pharmacy sell nutraceuticals? (Check sections or whether vitamins or other nutritional supplements are available)	yesno, Required<br>1 Yes<br>0 No
41	[indicat_use]	32. Does the e-pharmacy provide information on indication or use? Look for the following tracers ONLY. If the information is available even for one tracer, go with "Yes" amlodipine/nifedipine, atenolol/metoprolol, atorvastatin, metformin, amoxicillin, azithromycin, sildenafil/tadalafil	radio, Required<br>1 Yes<br>0 No<br>2 Incomplete
42	[side_effects]	33. Does the e-pharmacy provide information on drug's side effects? Look for the following tracers ONLY. If the information is available even for one tracer, go with "Yes" amlodipine/nifedipine, atenolol/metoprolol, atorvastatin, metformin, amoxicillin, azithromycin, sildenafil/tadalafil	yesno, Required<br>1 Yes<br>0 No
43	[drug_interactions]	34. Does the e-pharmacy provide information on drug interactions and contraindications? Look for the following tracers ONLY. If the information is available even for one tracer, go with "Yes" amlodipine/nifedipine, atenolol/metoprolol, atorvastatin, metformin, amoxicillin, azithromycin, sildenafil/tadalafil	radio, Required<br>1 Yes<br>0 No<br>2 Interactions only<br>3 Contraindications only
44	[online_consult]	35. Does the e-pharmacy offer online consultation with a doctor? (Check FAQ, "about", "services provided" or other relevant sections)	yesno, Required<br>1 Yes<br>0 No
45	[lab_services]	36. Does the e-pharmacy provide laboratory services? (Check FAQ, "about", "services provided" or other relevant sections)	yesno, Required<br>1 Yes<br>0 No

46	[prescription_upload]	37. Does e-pharmacy provide the option to upload prescription?	checkbox, Required<br>1 prescription_upload___1 Yes, on site<br>2 prescription_upload___2 Yes, WhatsApp<br>3 prescription_upload___3 Yes, email<br>4 prescription_upload___4 Other<br>5 prescription_upload___5 No
47	[other_prescription_upload]<br>Show the field ONLY if:<br>[prescription_upload(4)] = '1'	If other, please specify	text
48	[refill_remind]	38. Does the e-pharmacy state that they provide refill reminders?	yesno<br>1 Yes<br>0 No
49	[ad_pres_drugs]	Section Header: *Marketing Strategies*<br>39. Does the website have advertisements of any prescription drugs? (Check on the home page only)	yesno, Required<br>1 Yes<br>0 No
50	[ad_other_prod]	40. Does the website have advertisements of other products? (Check on the home page for ads on OTC creams/gels, sanitary pads etc)	yesno, Required<br>1 Yes<br>0 No
51	[discount_coupon]	41. Are there advertisements or announcements of discounts/coupons/offers? (Examine the advertisements, "offers" sections etc.)	yesno, Required<br>1 Yes<br>0 No
52	[discount_meds]<br>Show the field ONLY if:<br>[discount_coupon] = '1'	If yes, are there discounts/offers specifically for medicines? Use your judgment to determine whether offers apply to medicines or not.	yesno<br>1 Yes<br>0 No
53	[certi_accredi]	42. Does the e-pharmacy provide information on certifications/ accreditations assuring quality? (E.g., LegitScript, EU Logo, NABP accreditation, or similar)	yesno, Required<br>1 Yes<br>0 No
54	[certification_specify]<br>Show the field ONLY if:<br>[certi_accredi] = '1'	If yes, please specify the certification/accreditation:	text
55	[promo_social_media]	43. Does the e-pharmacy have social media presence? Run separate searches on Google (e.g., "Medplus Facebook" etc.) if information is unavailable on the website.	checkbox, Required<br>1 promo_social_media___1 Twitter<br>2 promo_social_media___2 Instagram<br>3 promo_social_media___3 Facebook<br>4 promo_social_media___4 TikTok<br>5 promo_social_media___5 Youtube<br>6 promo_social_media___6 LinkedIn<br>7 promo_social_media___7 Other<br>8 promo_social_media___8 None
56	[other_social]<br>Show the field ONLY if:<br>[promo_social_media(7)] = '1'	If other, please specify	text
57	[notes]	Additional Notes (Please write "na" if no additional notes)	notes
58	[e_pharmacy_website_complete]	Section Header: *Form Status*<br>Complete?	dropdown<br>0 Incomplete<br>1 Unverified<br>2 Complete
Instrument: **E Pharmacy App** (e_pharmacy_app)			
59	[e_pharm_name_app]<br>Show the field ONLY if:<br>[e_pharm_app]='1'	Section Header: *Basic Characteristics*<br>1. Name of the e-pharmacy (as mentioned on the site in lowercase without ".com")	text, Required, Identifier
60	[constitution_app]<br>Show the field ONLY if:<br>[e_pharm_app]='1'	2. Details of the e-pharmacy constitution (Information on directors/supervisors/chairmen/boards etc.)	yesno<br>1 Yes<br>0 No

61	[tel_number_app]<br><br>Show the field ONLY if:<br>[e_pharm_app]='1'	3. Telephone number of the e-pharmacy Capture office number. "0" if not provided.	text (number), Required, Identifier
62	[email_add_app]<br><br>Show the field ONLY if:<br>[e_pharm_app]='1'	4. Email address of the e-pharmacy "np" if not provided.	text, Required, Identifier
63	[phy_add_app]<br><br>Show the field ONLY if:<br>[e_pharm_app]='1'	5. Is there any physical address for the e-pharmacy?	yesno, Required<br>1 Yes<br>0 No
64	[sec_address_app]<br><br>Show the field ONLY if:<br>[e_pharm_app]='1'	6. Is there a second address?	yesno<br>1 Yes<br>0 No
65	[state_app]<br><br>Show the field ONLY if:<br>[e_pharm_app]='1'	7. State (Capture the state wherein the e-pharmacy's physical address is located in lowercase; separate multiple states with a comma without spaces. "np" if not provided.)	text, Required
66	[country_app]<br><br>Show the field ONLY if:<br>[e_pharm_app]='1'	8. Country If more than one country, capture all, separated by a comma without space.	text, Required
67	[store_app]<br><br>Show the field ONLY if:<br>[e_pharm_app]='1'	9. Which app stores can the e-pharmacy app be downloaded from?	checkbox, Required<br>1 store_app___1 Google Playstore<br>2 store_app___2 App Store (iOS)
68	[downloads_app]<br><br>Show the field ONLY if:<br>[store_app(1)] = '1'	Capture the number of downloads on Google Playstore. As provided in the PlayStore, e.g., 50M+ etc.	text
69	[authorization_info_app]<br><br>Show the field ONLY if:<br>[e_pharm_app]='1'	Section Header: *Authorization details*<br><br>10. Does the e-pharmacy provide information on authorization from the official licensing body (CDSCO for India and PPB for Kenya)? (refer to "about us" or "FAQ" pages of the e-pharmacy)	radio, Required<br>1 Yes, the app states that they are registered<br>2 Yes, the app states that they are registered and specifies the authorizing body<br>3 No
70	[reg_no_app]<br><br>Show the field ONLY if:<br>[e_pharm_app]='1'	11. Registration number or the health safety code of the e-pharmacy(E.g.: India: 3184/MIII/20, 3188/MIII/21, 5509/MIII/20B) "np" if not provided.	text, Required, Identifier
71	[pharmacist_details_app]<br><br>Show the field ONLY if:<br>[e_pharm_app]='1'	12. Does the e-pharmacy display the name and registration details of the pharmacist(s) involved? (refer to "about us" or "FAQ" pages of the e-pharmacy)	yesno, Required<br>1 Yes<br>0 No
72	[track_dlvry_app]<br><br>Show the field ONLY if:<br>[e_pharm_app]='1'	Section Header: *Customer Service*<br><br>13. Does the e-pharmacy app state that they provide any tracking of delivery? (Check FAQ or customer support sections)	yesno, Required<br>1 Yes<br>0 No
73	[lang_opt_app]<br><br>Show the field ONLY if:<br>[e_pharm_app]='1'	14. Are other language options present?	yesno, Required<br>1 Yes<br>0 No
74	[other_lang_app]<br><br>Show the field ONLY if:<br>[lang_opt_app] = '1'	If yes, please specify (Separate multiple languages with a comma without space)	text
75	[tele_helpline_app]<br><br>Show the field ONLY if:<br>[e_pharm_app]='1'	15. Is a telephonic helpline provided?	radio, Required<br>1 Yes, toll free<br>2 Yes, unspecified<br>3 Yes, paid<br>4 No
76	[chat_app]<br><br>Show the field ONLY if:<br>[e_pharm_app]='1'	16. Is chat option (live or bot) available for customers? Check homepage or "help" sections.	checkbox, Required<br>1 chat_app___1 Yes, on the app<br>2 chat_app___2 Yes on Whatsapp<br>3 chat_app___3 No

77	[faq_app]<br>Show the field ONLY if:<br>[e_pharm_app]='1'	17. Does the e-pharmacy provide an FAQ section? (Check "FAQ" or "Help")	yesno, Required<br>1 Yes<br>0 No
78	[complaints_app]<br>Show the field ONLY if:<br>[e_pharm_app]='1'	18. Does the e-pharmacy display the procedure for complaints in detail? (check FAQ, "complaints", or "contact us" pages, and copy paste the procedure for complaints)	yesno, Required<br>1 Yes<br>0 No
79	[review_testimonials_app]<br>Show the field ONLY if:<br>[e_pharm_app]='1'	19. Are customer reviews or testimonials visible on the e-pharmacy app?	yesno, Required<br>1 Yes<br>0 No
80	[return_policy_app]<br>Show the field ONLY if:<br>[e_pharm_app]='1'	20. Does the e-pharmacy display the return policy? (check FAQ, "refunds", or "contact us" pages)	yesno, Required<br>1 Yes<br>0 No
81	[privacy_policy_app]<br>Show the field ONLY if:<br>[e_pharm_app]='1'	Section Header: *Privacy*<br>21. Does the e-pharmacy display customers' privacy policy?	yesno, Required<br>1 Yes<br>0 No
82	[secur_encrypt_app]<br>Show the field ONLY if:<br>[e_pharm_app]='1'	22. Does the e-pharmacy display any certificates of security/encryption? (E.g., Kenya: Extended Validation SSL (EV-SSL) certificate)	yesno, Required<br>1 Yes<br>0 No
83	[encrypt_certif_app]<br>Show the field ONLY if:<br>[secur_encrypt_app] = '1'	If yes, please specify the certificate	text
84	[pay_options_app]<br>Show the field ONLY if:<br>[e_pharm_app]='1'	Section Header: *Payment*<br>23. Available payment options (choose all that apply)	checkbox, Required<br>1 pay_options_app___1 Credit Card<br>2 pay_options_app___2 Debit Card<br>3 pay_options_app___3 Net Banking<br>4 pay_options_app___4 Store Credit/Wallet<br>5 pay_options_app___5 Cash on delivery<br>6 pay_options_app___6 UPI<br>7 pay_options_app___7 Electronic wallet<br>8 pay_options_app___8 mPesa/Mobile Money<br>9 pay_options_app___9 PayPal<br>10 pay_options_app___10 Health insurance<br>11 pay_options_app___11 Others
85	[electronic_specify_app]<br>Show the field ONLY if:<br>[pay_options_app(7)] = '1'	If electronic wallet, please specify. (NR if not specified on the app)	text
86	[other_pay_app]<br>Show the field ONLY if:<br>[pay_options_app(11)] = '1'	If others, specify the payment method	text
87	[where_deliver_app]<br>Show the field ONLY if:<br>[e_pharm_app]='1'	Section Header: *Coverage*<br>24. Where all does the e-pharmacy deliver to?	radio, Required<br>1 Less than 50% of the Indian states<br>2 More than 50% of the Indian states<br>3 All over India<br>4 India and Neighbours<br>5 Global<br>6 Unclear<br>7 Other (please specify)
88	[other_geo_cover_app]<br>Show the field ONLY if:<br>[where_deliver_app] = '7'	If other, please specify the coverage	text
89	[otc_sale_app]<br>Show the field ONLY if:<br>[e_pharm_app]='1'	Section Header: *Pharmaceutical Aspects*<br>25. Are OTC medicines available for sale? Look for various OTC medicines, if even one OTC medicine is available FOR SALE, go with "Yes" Paracetamol, pantoprazole, diclofenac, cetirizine, clotrimazole	yesno, Required<br>1 Yes<br>0 No

90	[pom_sale_app]<br>Show the field ONLY if:<br>[e_pharm_app]='1'	26. Are prescription-only medicines available for sale? Look for various POMs, if even one POM is available FOR SALE, go with "Yes" e.g., Amlodipine, insulin, cefixime, atenolol, pregabalin, phenytoin	yesno, Required<br>1 Yes<br>0 No
91	[controlled_sale_app]<br>Show the field ONLY if:<br>[e_pharm_app]='1'	27. Are narcotic or controlled substances available for sale? Look for various narcotic or controlled drugs, if even one is available FOR SALE, go with "Yes" E.g., Tramadol, alprazolam, lorazepam, zolpidem, fentanyl	yesno<br>1 Yes<br>0 No
92	[herbal_altmed_app]<br>Show the field ONLY if:<br>[e_pharm_app]='1'	28. Does the e-pharmacy sell herbal/ alternative medicines? (Check whether "herbal products" or specific products such as arsenicum album, tulsi, aswagandha, or sarpagandha are available for sale)	yesno, Required<br>1 Yes<br>0 No
93	[nutra_app]<br>Show the field ONLY if:<br>[e_pharm_app]='1'	29. Does the e-pharmacy sell nutraceuticals? (Check sections or whether vitamins or other nutritional supplements are available)	yesno, Required<br>1 Yes<br>0 No
94	[indicat_use_app]<br>Show the field ONLY if:<br>[e_pharm_app]='1'	30. Does the e-pharmacy provide information on indication or use? Look for the following tracers ONLY. If the information is available even for one tracer, go with "Yes" amlodipine/nifedipine, atenolol/metoprolol, atorvastatin, metformin, amoxicillin, azithromycin, sildenafil/tadalafil	radio, Required<br>1 Yes<br>0 No<br>2 Incomplete
95	[side_effects_app]<br>Show the field ONLY if:<br>[e_pharm_app]='1'	31. Does the e-pharmacy provide information on drug's side effects? Look for the following tracers ONLY. If the information is available even for one tracer, go with "Yes" amlodipine/nifedipine, atenolol/metoprolol, atorvastatin, metformin, amoxicillin, azithromycin, sildenafil/tadalafil	yesno, Required<br>1 Yes<br>0 No
96	[drug_interactions_app]<br>Show the field ONLY if:<br>[e_pharm_app]='1'	32. Does the e-pharmacy provide information on drug interactions and contraindications? Look for the following tracers ONLY. If the information is available even for one tracer, go with "Yes" amlodipine/nifedipine, atenolol/metoprolol, atorvastatin, metformin, amoxicillin, azithromycin, sildenafil/tadalafil	radio, Required<br>1 Yes<br>0 No<br>2 Interactions only<br>3 Contraindications only
97	[online_consult_app]<br>Show the field ONLY if:<br>[e_pharm_app]='1'	33. Does the e-pharmacy offer online consultation with a doctor? (Check FAQ, "about", "services provided" or other relevant sections)	yesno, Required<br>1 Yes<br>0 No
98	[lab_services_app]<br>Show the field ONLY if:<br>[e_pharm_app]='1'	34. Does the e-pharmacy provide laboratory services? (Check FAQ, "about", "services provided" or other relevant sections)	yesno, Required<br>1 Yes<br>0 No
99	[app_prescription]<br>Show the field ONLY if:<br>[e_pharm_app] = '1'	35. Does e-pharmacy provide the option to upload prescription?	checkbox, Required<br>1 app_prescription___1 Yes, on app<br>2 app_prescription___2 Yes, WhatsApp<br>3 app_prescription___3 Yes, email<br>4 app_prescription___4 Other<br>5 app_prescription___5 No
100	[other_upload]<br>Show the field ONLY if:<br>[app_prescription(4)] = '1'	If other, please specify	text
101	[refill_remind_app]	36. Does the e-pharmacy state that they provide refill reminders?	yesno<br>1 Yes<br>0 No
102	[ad_pres_drugs_app]<br>Show the field ONLY if:<br>[e_pharm_app]='1'	Section Header: *Marketing Strategies*<br>37. Does the app have advertisements of any prescription drugs? (Check for pop-up advertisements on any pages during app review)	yesno, Required<br>1 Yes<br>0 No
103	[ad_other_prod_app]<br>Show the field ONLY if:<br>[e_pharm_app]='1'	38. Does the app have advertisements of other products? (Check for ads on OTC creams/gels, sanitary pads etc)	yesno, Required<br>1 Yes<br>0 No
104	[discount_coupon_app]<br>Show the field ONLY if:<br>[e_pharm_app]='1'	39. Are there advertisements or announcements of discounts/coupons/offers? (Examine the advertisements, "offers" sections etc.)	yesno, Required<br>1 Yes<br>0 No

105	[discount_meds_app]<br>Show the field ONLY if:<br>[discount_coupon_app] = '1'	If yes, are there discounts/offers specifically for medicines?	yesno<br>1 Yes<br>0 No
106	[certi_accredi_app]<br>Show the field ONLY if:<br>[e_pharm_app]='1'	40. Does the e-pharmacy provide information on certifications/ accreditations assuring quality? (E.g., LegitScript, EU Logo, NABP accreditation, or similar)	yesno, Required<br>1 Yes<br>0 No
107	[certification_specify_app]<br>Show the field ONLY if:<br>[certi_accredi_app] = '1'	If yes, please specify the certification/accreditation:	text
108	[notes_app]<br>Show the field ONLY if:<br>[e_pharm_app]='1'	Additional Notes (Please write "NA" if no additional notes)	notes
109	[e_pharmacy_app_complete]	Section Header: *Form Status*<br>Complete?	dropdown<br>0 Incomplete<br>1 Unverified<br>2 Complete

Data Dictionary Codebook

23-06-2023 10:10am

#	Variable / Field Name	Field Label<br>*Field Note*	Field Attributes (Field Type, Validation, Choices, Calculations, etc.)
**Instrument: E Pharmacy Website (e_pharmacy_website)**			
1	[record_id]	Record ID	text
2	[e_pharm_name]	Section Header: *Basic Characteristics*<br>1. Name of the e-pharmacy (as mentioned on the site in lowercase without ".com")	text, Required, Identifier
3	[web_add]	2. Complete web address (E.g.: https://www.netmeds.com/ or https://mydawa.com/)	text, Required, Identifier
4	[constitution]	3. Details of the e-pharmacy constitution (Information on directors/supervisors/chairmen/boards etc.)	yesno<br>1 Yes<br>0 No
5	[tel_number]	4. Telephone number of the e-pharmacy Capture office number. "0" if not provided.	text (number), Required, Identifier
6	[email_add]	5. Email address of the e-pharmacy "np" if not provided.	text, Required, Identifier
7	[phy_add]	6. Is there any physical address for the e-pharmacy?	yesno, Required<br>1 Yes<br>0 No
8	[sec_address]	7. Is there a second address?	yesno<br>1 Yes<br>0 No
9	[state]	8. County (Capture the county wherein the e-pharmacy's physical address is located in lowercase; separate multiple states with a comma without spaces. "np" if not provided.)	text, Required
10	[country]	9. Country If more than one country, capture all, separated by a comma without space.	text, Required
11	[authorization_info]	Section Header: *Authorization details*<br>10. Does the e-pharmacy provide information on authorization from the official licensing body (PPB for Kenya)? (refer to "about us" or "FAQ" pages of the e-pharmacy)	radio<br>1 Yes, the site states that they are registered<br>2 Yes, the site states that they are registered and specifies the authorizing body<br>3 No
12	[reg_no]	11. Registration number or the health safety code of the e-pharmacy(E.g.: mydawa: P0940)	text, Required, Identifier
13	[pharmacist_details]	12. Does the e-pharmacy display the name and registration details of the pharmacist(s) involved? (refer to "about us" or "FAQ" pages of the e-pharmacy)	yesno, Required<br>1 Yes<br>0 No
14	[e_pharm_app]	Section Header: *Business model*<br>13. Does the e-pharmacy have an app?	yesno, Required<br>1 Yes<br>0 No
15	[track_dlvry]	Section Header: *Customer Service*<br>14. Does the e-pharmacy website state that they provide any tracking of delivery? (Check FAQ or customer support sections)	yesno, Required<br>1 Yes<br>0 No
16	[lang_opt]	15. Are other language options present?	yesno, Required<br>1 Yes<br>0 No
17	[other_lang]<br>Show the field ONLY if:<br>[lang_opt] = '1'	If yes, please specify (Separate multiple languages with a comma without space)	text

18	[tele_helpline]	16. Is a telephonic helpline provided?	radio, Required<br>1 Yes, toll free<br>2 Yes, unspecified<br>3 Yes, paid<br>4 No
19	[chat]	17. Is chat option (live or bot) available for customers?	checkbox, Required<br>1 chat___1 Yes, on the website<br>2 chat___2 Yes on Whatsapp<br>3 chat___3 No
20	[faq]	18. Does the e-pharmacy provide an FAQ section? (Check "FAQ" or "Help")	yesno, Required<br>1 Yes<br>0 No
21	[complaints]	19. Does the e-pharmacy display the procedure for complaints in detail? (check FAQ, "complaints", or "contact us" pages, and copy paste the procedure for complaints)	yesno, Required<br>1 Yes<br>0 No
22	[complaint_url]<br>Show the field ONLY if:<br>[complaints] = '1'	If yes, please provide the URL.	text
23	[review_testimonials]	20. Are customer reviews or testimonials visible on the e-pharmacy website?	yesno, Required<br>1 Yes<br>0 No
24	[return_policy]	21. Does the e-pharmacy display the return policy? (check FAQ, "refunds", or "contact us" pages)	yesno, Required<br>1 Yes<br>0 No
25	[return_url]<br>Show the field ONLY if:<br>[return_policy] = '1'	If yes, please provide the URL.	text
26	[privacy_policy]	Section Header: *Privacy*<br>22. Does the e-pharmacy display customers' privacy policy?	yesno, Required<br>1 Yes<br>0 No
27	[privacy_url]<br>Show the field ONLY if:<br>[privacy_policy] = '1'	If yes, please provide the URL.	text
28	[secur_encrypt]	23. Does the e-pharmacy display any certificates of security/encryption? (E.g., Kenya: Extended Validation SSL (EV-SSL) certificate)	yesno, Required<br>1 Yes<br>0 No
29	[encrypt_certif]<br>Show the field ONLY if:<br>[secur_encrypt] = '1'	If yes, please specify the certificate	text
30	[pay_gateway]	Section Header: *Payment*<br>24. Is a secure payment gateway present? (Redirection to a separate page for payment)	radio, Required<br>1 Yes<br>0 No<br>2 Unclear

31	[pay_options]	25. Available payment options (choose all that apply)	checkbox, Required<br>1 pay_options___1 Credit Card<br>2 pay_options___2 Debit Card<br>3 pay_options___3 Net Banking<br>4 pay_options___4 Store Credit/Wallet<br>5 pay_options___5 Cash on delivery<br>6 pay_options___6 UPI<br>7 pay_options___7 Electronic wallet<br>8 pay_options___8 mPesa/Mobile Money<br>9 pay_options___9 PayPal<br>10 pay_options___10 Health insurance<br>11 pay_options___11 Others
32	[electronic_specify]<br>Show the field ONLY if:<br>[pay_options(7)] = '1'	If electronic wallet, please specify. ("np" if not specified on the website)	text
33	[other_pay]<br>Show the field ONLY if:<br>[pay_options(11)] = '1'	If others, specify the payment method	text
34	[where_deliver]	Section Header: *Coverage*<br>26. Where all does the e-pharmacy deliver to?	radio, Required<br>1 Less than 50% of counties in Kenya<br>2 More than 50% of counties in Kenya<br>3 All over Kenya<br>4 Kenya and Neighbours<br>5 Global<br>6 Unclear<br>7 Other (please specify)
35	[other_geo_cover]<br>Show the field ONLY if:<br>[where_deliver] = '7'	If other, please specify the coverage	text
36	[otc_sale]	Section Header: *Pharmaceutical Aspects*<br>27. Are OTC medicines available for sale? Look for various OTC medicines, if even one OTC medicine is available FOR SALE, go with "Yes" E.g., Paracetamol, pantoprazole, diclofenac, cetirizine, clotrimazole	yesno, Required<br>1 Yes<br>0 No
37	[pom_sale]	28. Are prescription-only medicines available for sale? Look for various POMs, if even one POM is available FOR SALE, go with "Yes" e.g., Amlodipine, insulin, cefixime, atenolol, pregabalin, phenytoin	yesno, Required<br>1 Yes<br>0 No
38	[controlled_sale]	29. Are narcotic or controlled substances available for sale? Look for various narcotic or controlled drugs, if even one is available FOR SALE, go with "Yes" E.g., Tramadol, alprazolam, lorazepam, zolpidem, fentanyl	yesno, Required<br>1 Yes<br>0 No
39	[herbal_altmed]	30. Does the e-pharmacy sell herbal/ alternative medicines? (Check whether "herbal products" or specific products such as arsenicum album, tulsi, aswagandha, or sarpagandha are available for sale)	yesno, Required<br>1 Yes<br>0 No
40	[nutra]	31. Does the e-pharmacy sell nutraceuticals? (Check sections or whether vitamins or other nutritional supplements are available)	yesno, Required<br>1 Yes<br>0 No
41	[indicat_use]	32. Does the e-pharmacy provide information on indication or use? Look for the following tracers ONLY. If the information is available even for one tracer, go with "Yes" amlodipine/nifedipine, atenolol/metoprolol, atorvastatin, metformin, amoxicillin, azithromycin, sildenafil/tadalafil	radio, Required<br>1 Yes<br>0 No<br>2 Incomplete
42	[side_effects]	33. Does the e-pharmacy provide information on drug's side effects? Look for the following tracers ONLY. If the information is available even for one tracer, go with "Yes" amlodipine/nifedipine, atenolol/metoprolol, atorvastatin, metformin, amoxicillin, azithromycin, sildenafil/tadalafil	yesno, Required<br>1 Yes<br>0 No

43	[drug_interactions]	34. Does the e-pharmacy provide information on drug interactions and contraindications? Look for the following tracers ONLY. If the information is available even for one tracer, go with "Yes" amlodipine/nifedipine, atenolol/metoprolol, atorvastatin, metformin, amoxicillin, azithromycin, sildenafil/tadalafil	radio, Required<br>1 Yes<br>0 No<br>2 Interactions only<br>3 Contraindications only
44	[online_consult]	35. Does the e-pharmacy offer online consultation with a doctor? (Check FAQ, "about", "services provided" or other relevant sections)	yesno, Required<br>1 Yes<br>0 No
45	[lab_services]	36. Does the e-pharmacy provide laboratory services? (Check FAQ, "about", "services provided" or other relevant sections)	yesno, Required<br>1 Yes<br>0 No
46	[prescription_upload]	37. Does e-pharmacy provide the option to upload prescription?	checkbox, Required<br>1 prescription_upload___1 Yes, on site<br>2 prescription_upload___2 Yes, WhatsApp<br>3 prescription_upload___3 Yes, email<br>4 prescription_upload___4 Other<br>5 prescription_upload___5 No
47	[prescription_other]<br>Show the field ONLY if:<br>[prescription_upload(4)] = '1'	If other, please specify	text
48	[refill_reminder]	38. Does the e-pharmacy state that they provide refill reminders?Check home page, FAQ, and any other relevant sections.	radio<br>1 Yes<br>0 No
49	[ad_pres_drugs]	Section Header: *Marketing Strategies*<br>39. Does the website have advertisements of any prescription drugs? (Check on the home page only)	yesno, Required<br>1 Yes<br>0 No
50	[ad_other_prod]	40. Does the website have advertisements of other products? (Check on the home page for ads on OTC creams/gels, sanitary pads etc)	yesno, Required<br>1 Yes<br>0 No
51	[discount_coupon]	41. Are there advertisements or announcements of discounts/coupons/offers? (Examine the advertisements, "offers" sections etc.)	yesno, Required<br>1 Yes<br>0 No
52	[discount_meds]<br>Show the field ONLY if:<br>[discount_coupon] = '1'	If yes, are there discounts/offers specifically for medicines? Use your judgment to determine whether offers apply to medicines or not.	yesno<br>1 Yes<br>0 No
53	[certi_accredi]	42. Does the e-pharmacy provide information on certifications/ accreditations assuring quality? (E.g., LegitScript, EU Logo, NABP accreditation, or similar)	yesno, Required<br>1 Yes<br>0 No
54	[certification_specify]<br>Show the field ONLY if:<br>[certi_accredi] = '1'	If yes, please specify the certification/accreditation:	text
55	[promo_social_media]	43. Does the e-pharmacy have social media presence? Run separate searches on Google (e.g., "Medplus Facebook" etc.) if information is unavailable on the website.	checkbox, Required<br>1 promo_social_media___1 Twitter<br>2 promo_social_media___2 Instagram<br>3 promo_social_media___3 Facebook<br>4 promo_social_media___4 TikTok<br>5 promo_social_media___5 Youtube<br>6 promo_social_media___6 LinkedIn<br>7 promo_social_media___7 Other<br>8 promo_social_media___8 None
56	[other_social]<br>Show the field ONLY if:<br>[promo_social_media(7)] = '1'	If other, please specify	text
57	[notes]	Additional Notes (Please write "NA" if no additional notes)	notes

58	[e_pharmacy_website_complete]	Section Header: *Form Status*<br>Complete?	dropdown<br>0 Incomplete<br>1 Unverified<br>2 Complete
**Instrument: E Pharmacy App (e_pharmacy_app)**			
59	[e_pharm_name_app]<br>Show the field ONLY if:<br>[e_pharm_app] = '1'	Section Header: *Basic Characteristics*<br>1. Name of the e-pharmacy (as mentioned on the app in lowercase without ".com")	text, Required, Identifier
60	[constitution_app]<br>Show the field ONLY if:<br>[e_pharm_app] = '1'	2. Details of the e-pharmacy constitution (Information on directors/supervisors/chairmen/boards etc.)	yesno<br>1 Yes<br>0 No
61	[tel_number_app]<br>Show the field ONLY if:<br>[e_pharm_app] = '1'	3. Telephone number of the e-pharmacy Capture office number. "0" if not provided.	text (number), Required, Identifier
62	[email_add_app]<br>Show the field ONLY if:<br>[e_pharm_app] = '1'	4. Email address of the e-pharmacy "np" if not provided.	text, Required, Identifier
63	[phy_add_app]<br>Show the field ONLY if:<br>[e_pharm_app] = '1'	5. Is there any physical address for the e-pharmacy?	yesno, Required<br>1 Yes<br>0 No
64	[sec_address_app]<br>Show the field ONLY if:<br>[e_pharm_app] = '1'	6. Is there a second address?	yesno<br>1 Yes<br>0 No
65	[state_app]<br>Show the field ONLY if:<br>[e_pharm_app] = '1'	7. County (Capture the county wherein the e-pharmacy's physical address is located in lowercase; separate multiple states with a comma without spaces. "np" if not provided.)	text, Required
66	[country_app]<br>Show the field ONLY if:<br>[e_pharm_app] = '1'	8. Country If more than one country, capture all, separated by a comma without space.	text, Required
67	[store_app]<br>Show the field ONLY if:<br>[e_pharm_app] = '1'	9. Which app stores can the e-pharmacy app be downloaded from?	checkbox, Required<br>1 store_app___1 Google Playstore<br>2 store_app___2 App Store (iOS)
68	[downloads_app]<br>Show the field ONLY if:<br>[store_app(1)] = '1'	Capture the number of downloads on Google Playstore. (e.g., 50M+, 500K+)	text
69	[authorization_info_app]<br>Show the field ONLY if:<br>[e_pharm_app] = '1'	Section Header: *Authorization details*<br>10. Does the e-pharmacy provide information on authorization from the official licensing body (PPB for Kenya)? (refer to "about us" or "FAQ" pages of the e-pharmacy)	radio, Required<br>1 Yes, the app states that they are registered<br>2 Yes, the app states that they are registered and specifies the authorizing body<br>3 No
70	[reg_no_app]<br>Show the field ONLY if:<br>[e_pharm_app] = '1'	11. Registration number or the health safety code of the e-pharmacy(E.g.: mydawa: P0940)	text, Required, Identifier
71	[pharmacist_details_app]<br>Show the field ONLY if:<br>[e_pharm_app] = '1'	12. Does the e-pharmacy display the name and registration details of the pharmacist(s) involved? (refer to "about us" or "FAQ" pages of the e-pharmacy)	yesno, Required<br>1 Yes<br>0 No
72	[track_dlvry_app]<br>Show the field ONLY if:<br>[e_pharm_app] = '1'	Section Header: *Customer Service*<br>13. Does the e-pharmacy app state that they provide any tracking of delivery? (Check FAQ or customer support sections)	yesno, Required<br>1 Yes<br>0 No
73	[lang_opt_app]<br>Show the field ONLY if:<br>[e_pharm_app] = '1'	14. Are other language options present?	yesno, Required<br>1 Yes<br>0 No
74	[other_lang_app]<br>Show the field ONLY if:<br>[lang_opt_app] = '1'	If yes, please specify (Separate multiple languages with a comma without space)	text

75	[tele_helpline_app]<br>Show the field ONLY if:<br>[e_pharm_app] = '1'	15. Is a telephonic helpline provided?	radio, Required<br>1 Yes, toll free<br>2 Yes, unspecified<br>3 No
76	[chat_app]<br>Show the field ONLY if:<br>[e_pharm_app] = '1'	16. Is chat option (live or bot) available for customers?	checkbox, Required<br>1 chat_app___1 Yes, on the app<br>2 chat_app___2 Yes on Whatsapp<br>3 chat_app___3 No
77	[faq_app]<br>Show the field ONLY if:<br>[e_pharm_app] = '1'	17. Does the e-pharmacy provide an FAQ section? (Check "FAQ" or "Help")	yesno, Required<br>1 Yes<br>0 No
78	[complaints_app]<br>Show the field ONLY if:<br>[e_pharm_app] = '1'	18. Does the e-pharmacy display the procedure for complaints in detail? (check FAQ, "complaints", or "contact us" pages, and copy paste the procedure for complaints)	yesno, Required<br>1 Yes<br>0 No
79	[review_testimonials_app]<br>Show the field ONLY if:<br>[e_pharm_app] = '1'	19. Are customer reviews or testimonials visible on the e-pharmacy app?	yesno, Required<br>1 Yes<br>0 No
80	[return_policy_app]<br>Show the field ONLY if:<br>[e_pharm_app] = '1'	20. Does the e-pharmacy display the return policy? (check FAQ, "refunds", or "contact us" pages)	yesno, Required<br>1 Yes<br>0 No
81	[privacy_policy_app]<br>Show the field ONLY if:<br>[e_pharm_app] = '1'	Section Header: *Privacy*<br>21. Does the e-pharmacy display customers' privacy policy?	yesno, Required<br>1 Yes<br>0 No
82	[secur_encrypt_app]<br>Show the field ONLY if:<br>[e_pharm_app] = '1'	22. Does the e-pharmacy display any certificates of security/encryption? (E.g., Kenya: Extended Validation SSL (EV-SSL) certificate)	yesno<br>1 Yes<br>0 No
83	[encrypt_certif_app]<br>Show the field ONLY if:<br>[secur_encrypt_app] = '1'	If yes, please specify the certificate	text
84	[pay_options_app]<br>Show the field ONLY if:<br>[e_pharm_app] = '1'	Section Header: *Payment*<br>23. Available payment options (choose all that apply)	checkbox, Required<br>1 pay_options_app___1 Credit Card<br>2 pay_options_app___2 Debit Card<br>3 pay_options_app___3 Net Banking<br>4 pay_options_app___4 Store Credit/Wallet<br>5 pay_options_app___5 Cash on delivery<br>6 pay_options_app___6 UPI<br>7 pay_options_app___7 Electronic wallet<br>8 pay_options_app___8 mPesa/Mobile Money<br>9 pay_options_app___9 PayPal<br>10 pay_options_app___10 Health insurance<br>11 pay_options_app___11 Others
85	[electronic_specify_app]<br>Show the field ONLY if:<br>[pay_options_app(7)] = '1'	If electronic wallet, please specify. ("np" if not specified on the app)	text
86	[other_pay_app]<br>Show the field ONLY if:<br>[pay_options_app(11)] = '1'	If others, specify the payment method	text

87	[where_deliver_app]<br>Show the field ONLY if:<br>[e_pharm_app] = '1'	Section Header: *Coverage*<br>24. Where all does the e-pharmacy deliver to?	radio, Required<br>1 Less than 50% of the counties of Kenya<br>2 More than 50% of the counties of Kenya<br>3 All over Kenya<br>4 Kenya and Neighbours<br>5 Global<br>6 Unclear<br>7 Other (please specify)
88	[other_geo_cover_app]<br>Show the field ONLY if:<br>[where_deliver_app] = '7'	If other, please specify the coverage	text
89	[otc_sale_app]<br>Show the field ONLY if:<br>[e_pharm_app] = '1'	Section Header: *Pharmaceutical Aspects*<br>25. Are OTC medicines available for sale? Look for various OTC medicines, if even one OTC medicine is available FOR SALE, go with "Yes" E.g., Paracetamol, pantoprazole, diclofenac, cetirizine, clotrimazole	yesno, Required<br>1 Yes<br>0 No
90	[pom_sale_app]<br>Show the field ONLY if:<br>[e_pharm_app] = '1'	26. Are prescription-only medicines available for sale? Look for various POMs, if even one POM is available FOR SALE, go with "Yes" e.g., Amlodipine, insulin, cefixime, atenolol, pregabalin, phenytoin	yesno, Required<br>1 Yes<br>0 No
91	[controlled_sale_app]<br>Show the field ONLY if:<br>[e_pharm_app] = '1'	27. Are narcotic or controlled substances available for sale? Look for various narcotic or controlled drugs, if even one is available FOR SALE, go with "Yes" E.g., Tramadol, alprazolam, lorazepam, zolpidem, fentanyl	yesno, Required<br>1 Yes<br>0 No
92	[herbal_altmed_app]<br>Show the field ONLY if:<br>[e_pharm_app] = '1'	28. Does the e-pharmacy sell herbal/ alternative medicines? (Check whether "herbal products" or specific products such as arsenicum album, tulsi, aswagandha, or sarpagandha are available for sale)	yesno, Required<br>1 Yes<br>0 No
93	[nutra_app]<br>Show the field ONLY if:<br>[e_pharm_app] = '1'	29. Does the e-pharmacy sell nutraceuticals? (Check sections or whether vitamins or other nutritional supplements are available)	yesno, Required<br>1 Yes<br>0 No
94	[indicat_use_app]<br>Show the field ONLY if:<br>[e_pharm_app] = '1'	30. Does the e-pharmacy provide information on indication or use? Look for the following tracers ONLY. If the information is available even for one tracer, go with "Yes" amlodipine/nifedipine, atenolol/metoprolol, atorvastatin, metformin, amoxicillin, azithromycin, sildenafil/tadalafil	radio, Required<br>1 Yes<br>0 No<br>2 Incomplete
95	[side_effects_app]<br>Show the field ONLY if:<br>[e_pharm_app] = '1'	31. Does the e-pharmacy provide information on drug's side effects? Look for the following tracers ONLY. If the information is available even for one tracer, go with "Yes" amlodipine/nifedipine, atenolol/metoprolol, atorvastatin, metformin, amoxicillin, azithromycin, sildenafil/tadalafil	yesno, Required<br>1 Yes<br>0 No
96	[drug_interactions_app]<br>Show the field ONLY if:<br>[e_pharm_app] = '1'	32. Does the e-pharmacy provide information on drug interactions and contraindications? Look for the following tracers ONLY. If the information is available even for one tracer, go with "Yes" amlodipine/nifedipine, atenolol/metoprolol, atorvastatin, metformin, amoxicillin, azithromycin, sildenafil/tadalafil	radio, Required<br>1 Yes<br>0 No<br>2 Interactions only<br>3 Contraindications only
97	[online_consult_app]<br>Show the field ONLY if:<br>[e_pharm_app] = '1'	33. Does the e-pharmacy offer online consultation with a doctor? (Check FAQ, "about", "services provided" or other relevant sections)	yesno, Required<br>1 Yes<br>0 No
98	[lab_services_app]<br>Show the field ONLY if:<br>[e_pharm_app] = '1'	34. Does the e-pharmacy provide laboratory services? (Check FAQ, "about", "services provided" or other relevant sections)	yesno, Required<br>1 Yes<br>0 No
99	[prescription_app_upload]<br>Show the field ONLY if:<br>[e_pharm_app] = '1'	35. Does e-pharmacy provide the option to upload prescription?	checkbox, Required<br>1 prescription_app_upload___1 Yes, on app<br>2 prescription_app_upload___2 Yes, WhatsApp<br>3 prescription_app_upload___3 Yes, email<br>4 prescription_app_upload___4 Other<br>5 prescription_app_upload___5 No

100	[prescription_other_app]<br>Show the field ONLY if:<br>[e_pharm_app] = '1'	If other, please specify	text
101	[refill_remind_app]	36. Does the e-pharmacy state that they provide refill reminders? Check home page, FAQ, and any other relevant sections.	radio<br>1 Yes<br>0 No
102	[ad_pres_drugs_app]<br>Show the field ONLY if:<br>[e_pharm_app] = '1'	Section Header: *Marketing Strategies*<br>37. Does the app have advertisements of any prescription drugs? (Check for pop-up advertisements on any pages during app review)	yesno, Required<br>1 Yes<br>0 No
103	[ad_other_prod_app]<br>Show the field ONLY if:<br>[e_pharm_app] = '1'	38. Does the app have advertisements of other products? (Check for ads on OTC creams/gels, sanitary pads etc)	yesno, Required<br>1 Yes<br>0 No
104	[discount_coupon_app]<br>Show the field ONLY if:<br>[e_pharm_app] = '1'	39. Are there advertisements or announcements of discounts/coupons/offers? (Examine the advertisements, "offers" sections etc.)	yesno, Required<br>1 Yes<br>0 No
105	[discount_meds_app]<br>Show the field ONLY if:<br>[discount_coupon_app] = '1'	If yes, are there discounts/offers specifically for medicines?	yesno<br>1 Yes<br>0 No
106	[certi_accredi_app]<br>Show the field ONLY if:<br>[e_pharm_app] = '1'	40. Does the e-pharmacy provide information on certifications/ accreditations assuring quality? (E.g., LegitScript, EU Logo, NABP accreditation, or similar)	yesno, Required<br>1 Yes<br>0 No
107	[certification_specify_app]<br>Show the field ONLY if:<br>[certi_accredi_app] = '1'	If yes, please specify the certification/accreditation:	text
108	[notes_app]<br>Show the field ONLY if:<br>[e_pharm_app] = '1'	Additional Notes (Please write "NA" if no additional notes)	notes
109	[e_pharmacy_app_complete]	Section Header: *Form Status*<br>Complete?	dropdown<br>0 Incomplete<br>1 Unverified<br>2 Complete
