# Supplementary material for: The good, the bad, and the ugly: Compliance of e-pharmacies serving India and Kenya with regulatory requirements and best practices
Source: PLOS Glob Public Health. 2025 Feb 3;5(2):e0004202. doi: 10.1371/journal.pgph.0004202 (PMC11790122; doi:10.1371/journal.pgph.0004202)
Supplement: S2 Table — (DOCX) [file pgph.0004202.s003.docx]

**SUPPLEMENTARY FILE**

**S2 Table:** E-pharmacy characteristics and business practices for websites with associated apps only, n (%)

|  | India | | Kenya | |
| --- | --- | --- | --- | --- |
|  | Websites | Apps | Websites | Apps |
| Number of e-pharmacy websites with apps reviewed (N) | **33** | | **3** | |
| Products displayed for sale  OTC medicines  TCAM medicines  Nutraceuticals | 31 (93.9)  30 (90.9)  31 (93.9) | 31 (93.9)  30 (90.9)  31 (93.9) | 3 (100)  3 (100)  3 (100) | 3 (100.0)  3 (100.0)  3 (100.0) |
| Additional services provided  Online consultation with a doctor  Laboratory tests  Both  Neither (pharmacy only) | 15 (45.4)  12 (36.4)  10 (30.3)  16 (48.4) | 15 (45.4)  14 (42.4)  10 (30.3)  14 (42.4) | 1 (33.3)  0 (0.0)  0 (0.0)  2 (66.7) | 1 (33.3)  0 (0.0)  0 (0.0)  2 (66.7) |
| Coverage of services  Delivers only to specific states/counties  Delivers anywhere within the country  Delivers globally  Unclear | 14 (42.4)  9 (27.3)  2 (6.1)  8 (24.2) | 12 (36.4)  7(21.2)  1 (3.0)  13 (39.4) | 1 (33.3)  2 (66.7)  0 (0.0)  0 (0.0) | 1 (33.3)  2 (66.7)  0 (0.0)  0 (0.0) |
| Marketing strategies  Announces offers or discounts^1^ | 31 (93.9) | 28 (84.8) | 2 (66.7) | 3 (100.0) |
| Customer service  FAQ section  Additional language options^+^  Customer reviews/testimonials | 24 (72.7)  3 (9.1)  11 (33.3) | 20 (60.6)  4 (12.1)  5 (15.1) | 3 (100.0)  0 (0.0)  2 (66.7) | 3 (100.0)  0 (0.0)  1 (33.3) |
| Payment Modes  *Credit/Debit Card*  *Net Banking*  *Store Credit*  *UPI*  *Electronic wallet*  *Cash on Delivery*  *Mobile money*  *Bitcoin or crypto*  *Other*  *Unclear* | 21 (63.6)  23 (69.7)  5 (15.1)  24 (72.7)  19 (57.6)  26 (78.8)  0 (0.0)  0 (0.0)  9 (27.3)  3 (9.1) | 23 (69.7)  26 (78.8)  5 (15.1)  25 (75.8)  20 (60.6)  26 (78.8)  0 (0.0)  0 (0.0)  10 (30.3)  2 (6.1) | 2 (66.7)  1 (33.3)  1 (33.3)  0 (0.0)  1 (33.3)  1 (33.3)  3 (100.0)  0 (0.0)  2 (66.7)  0 (0.0) | 1 (33.3)  1 (33.3)  0 (0.0)  0 (0.0)  0 (0.0)  0 (0.0)  3 (100.0)  0 (0.0)  2 (66.7)  0 (0.0) |
| ^1^ We checked for discounts on offers pages and the homepage only. ^+^Additional language options included a mixture of regional [e.g., Hindi, Bangla, etc.] and other national languages [e.g., French, Spanish, etc.].  FAQ: Frequently asked questions; UPI: Unified Payments Interface; OTC: over the counter; TCAM: Traditional, complementary and alternative medicine; POM: Prescription only medicine. | | | | |
