# Supplementary material for: The good, the bad, and the ugly: Compliance of e-pharmacies serving India and Kenya with regulatory requirements and best practices
Source: PLOS Glob Public Health. 2025 Feb 3;5(2):e0004202. doi: 10.1371/journal.pgph.0004202 (PMC11790122; doi:10.1371/journal.pgph.0004202)
Supplement: S3 Table — (DOCX) [file pgph.0004202.s004.docx]

**SUPPLEMENTARY FILE**

**S2 Table:** Compliance with best practices for websites with associated apps only, n(%)

|  | India | | Kenya | |
| --- | --- | --- | --- | --- |
|  | Websites | Apps | Websites | Apps |
| Total number of e-pharmacy websites with apps reviewed (N) | **33** | | **3** | |
| Information provided on medicines^1^  *Indications*  *Side-effects*  *Drug interactions*  *Contraindications*  *All the above* | 20 (60.6)  20 (60.6)  11 (33.3)  11 (33.3)  7 (21.2) | 19 (57.6)  19 (57.6)  9 (27.3)  10 (30.3)  7 (21.2) | 1 (33.3)  1 (33.3)  1 (33.3)  1 (33.3)  1 (33.3) | 1 (33.3)  1 (33.3)  1 (33.3)  1 (33.3)  1 (33.3) |
| Option to upload a prescription  *On website/app*  *On WhatsApp*  *Via e-mail*  *Others (fax, post etc.)*  *Unavailable* | 32 (97.0)  4 (12.1)  2 (6.1)  0 (0.0)  1 (3.0) | 33 (100.0)  4 (12.1)  1 (3.0)  0 (0.0)  0 (0.0) | 2 (66.7)  0 (0.0)  0 (0.0)  0 (0.0)  1 (33.3) | 1 (33.3)  0 (0.0)  0 (0.0)  0 (0.0)  2 (66.7) |
| Does not display narcotics or controlled substances for sale | 22 (66.7) | 21 (63.6) | 1 (33.3) | 1 (33.3) |
| Does not display product-specific advertisements for POMs | 37 (100.0) | 37 (100.0) | 3 (100.0) | 3 (100.0) |
| Provides refill reminders | 6 (18.2) | 13 (40.6) | 0 (0.0) | 0 (0.0) |
| Provides a complete physical address | 31 (93.9) | 22 (66.7) | 3 (100.0) | 3 (100.0) |
| Provides a telephonic helpline | 30 (90.9) | 25 (75.8) | 3 (100.0) | 3 (100.0) |
| Provides assistance via chat  *On website/app*  *On WhatsApp* | 8 (24.2)  7 (21.2) | 10 (30.3)  9 (27.3) | 2 (66.7)  3 (100.0) | 1 (33.3)  1 (33.3) |
| Provides tracking of delivery | 21 (63.6) | 18 (54.6) | 2 (66.7) | 2 (66.7) |
| Provides registration details of the e-pharmacy | 1 (3.0) | 0 (0.0) | 1 (33.3) | 1 (33.3) |
| Provides name and details of the pharmacy director/ superintendent/ owner | 10 (30.3) | 5 (15.1) | 0 (0.0) | 0 (0.0) |
| Provides registration details of the pharmacist(s) involved | 0 (0.0) | 0 (0.0) | 0 (0.0) | 1 (33.3) |
| Displays customers’ privacy policy | 32 (97.0) | 28 (84.8) | 3 (100.0) | 3 (100.0) |
| Displays a detailed procedure for grievance redressal | 9 (27.3) | 13 (39.4) | 0 (0.0) | 0 (0.0) |
| ^1^ This information was assessed based on selected ‘tracer medicines’, which include some of the most commonly sold prescription-only medicines in India and Kenya. | | | | |
