# Supplementary material for: The good, the bad, and the ugly: Compliance of e-pharmacies serving India and Kenya with regulatory requirements and best practices
Source: PLOS Glob Public Health. 2025 Feb 3;5(2):e0004202. doi: 10.1371/journal.pgph.0004202 (PMC11790122; doi:10.1371/journal.pgph.0004202)
Supplement: S2 File — (PDF) [file pgph.0004202.s005.pdf]

PRA/LPP/GUD/033

Revision No. 01

REPUBLIC OF KENYA

MINISTRY OF HEALTH

PHARMACY AND POISONS BOARD

GUIDELINES FOR INTERNET PHARMACY SERVICES IN KENYA

NOVEMBER 2022

Citation and Address

The author of this document is the Pharmacy and Poisons Board, a State Corporation under the Ministry of Health, Republic of Kenya. It may be reproduced or adopted on condition that the recommended citation below is made.

Recommended citation: *Republic of Kenya, Ministry of Health, Pharmacy and Poisons Board, Guidelines for Internet Pharmacy Services in Kenya Version 1, 2022*

©Pharmacy and Poisons Board, 2022

All rights reserved:

No part of this publication may be reproduced, stored in a retrieval system, or transmitted in any form, or by any means, electronic, mechanical, photocopying, recording, scanning, or otherwise, without the prior permission in writing from the pharmacy and Poisons Board.

For clarifications, comments, or suggestions, please contact:

The Chief Executive Officer
Pharmacy and Poisons Board
P.O. Box 27663 – 00506, Nairobi
Telephone: 0709770100
Email: info@pharmacyboardkenya.org
Website: www.pharmacyboardkenya.org

ORIGINAL COPY
23 NOV 2022
Sign:...for...........................
PHARMACY AND POISONS BOARD

Prepared by:

Deputy Director,

Sign...

Date....22/11/2022...

Reviewed by:

Director,

Sign...

Date....23/11/2022...

Checked by:

Head, Quality Management

Sign...

Date....23/11/2022...

Authorized by:

Chief Executive Officer/Registrar

Dr. Fred Siyoi

Sign...

Date....23/11/20[illegible]...

Table of Contents

Table of Contents

Abbreviations and Acronyms iv
Glossary of Terms v
Acknowledgements vii
1. Introduction 2
Background 2
Legal framework 3
Scope 3
2. Requirements for Internet Pharmacy Services in Kenya 4
Registration of Internet pharmacy services 4
3. Minimum Standards for Pharmacies Operating Internet Sites 5
4. Professional Services 8
5. Dispensing 8
6. Information and advice 9
7. Delivery of medicines 10
8. Record keeping 11
9. Security and confidentiality 12
10. Website requirements 12
11. Technology Equipment and Software 13
12. Quality Assurance 14
13. Requirements for online Pharmacy 15
References 18
Annexes 19
Annex 1: Online Pharmacy Premises Approval Flowchart 19

Abbreviations and Acronyms

GDP	Good distribution practices
GMP	Good manufacturing practices
PPB	Pharmacy and Poisons Board
ICT	Information Communication Technology

Glossary of Terms

**Enrolled pharmaceutical technologist**	A pharmaceutical technologist whose name appears on the roll.
**Internet Pharmacy**	A registered pharmacy which offers to sell or supply medicines (or other pharmaceutical products) and/or provides other pharmaceutical professional services over the internet, or plans for the supply of such products or provision of such services over the internet
**Health Products and Technologies**	Medicines, vaccines, blood and blood products and medical devices, including in-vitro diagnostics, food supplements, cosmetics, radiopharmaceuticals, cells, tissues and organs, complementary and alternative medicines, and borderline products.
**Pharmacy Business**	where the dispensing and compounding of prescriptions for any substance specified as a Part 1 poison in the Poisons List proclaimed under the Pharmacy and Poisons Act occurs
**Premises**	A fixed portion of any building, structure or vessel leased, used, or controlled by the licensee in the conduct of the pharmacy business registered by the Board at the address for which the registration was issued under section 23 of the Pharmacy and Poisons Act and includes all those areas where medicinal products are, or are intended to be, sold, or supplied, prepared, dispensed, compounded, or stored
**Registered pharmacist**	A person whose name is entered in the register.

**Registered pharmacy**	Any retail pharmacy premises and shall include, in cases where e-pharmacy or an online pharmacy practice has been licensed, the premises where the practice is domiciled
**Registered premises**	Premises registered in accordance with section 23 of the Pharmacy and Poisons Act, and where a valid certificate for registration is available;
**Superintendent**	The duly qualified and authorized person who is overall in charge of ensuring regulatory compliance.

Acknowledgements

The Pharmacy and Poisons Board wishes to express its appreciation to all whose efforts and valuable contributions in developing this guideline on registration and licensing of premises.

The PPB gratefully acknowledges the contributions of the following persons who contributed to the guidelines:

Dr. Fred Siyoi	CEO, Pharmacy and Poisons Board
Dr. Wilfred O. Oguta	Director, Pharmacy Practice & Training, PPB
Dr. Dominic Mutie	Deputy Director, Licensing and GPP, PPB
Dr. Humphrey Mwavali	Head, Registration & Enrolment, PPB
Dr. Abdulkadir Omar	Head, Licensing, PPB
Dr. Lily Kipkeno	Head, Training and CPD, PPB
Mr. George Muthuri	Registration & Enrolment, PPB

The Board also wishes to thank the USAID PQM+ Program, implemented by USP, for the financial and technical support offered in developing this guideline.

Finally, the Board also wishes to thank all PPB staff for their contribution, dedicated work, and coordination in the publication of these guidelines

Preface

The Pharmacy and Poisons Board is the National Medicines' Regulatory Authority established under the Pharmacy and Poisons Act, Chapter 244 of the Laws of Kenya. The Board is mandated to regulate the profession of Pharmacy, the manufacture and trade in medical products and health technologies. The overall goal is to maintain the required level of oversight while facilitating innovation and access to safe, effective, and good quality medical products and professionals that are fit to practice.

Pursuant to the Pharmacy and Poisons Act, a person cannot carry on a pharmacy business in Kenya unless the premises have a current approval and all the holders of the financial interest in the pharmacy business are registered as pharmacists or enrolled as pharmaceutical technologists.

With advancement in information technology and fast-growing access to the internet and smart mobile phones, there has been significant change in consumer behavior and demand for online services. Pharmaceutical services have not been left out and demand for internet pharmacy (also referred to as online pharmacy) services grew tremendously during the Covid-19 pandemic.

These Guidelines are intended to provide guidance to entities that offer access to health products and technologies through the internet and the public on registration and licensing of the business. It aims to highlight the minimum requirements for licensing and operations.

The guidelines are also intended to address challenges in the current practice environment, enhance implementation of current legislation, align licensing requirements with other PPB guidelines and respond to changes in technology.

Dr. F.M. Siyoi
CHIEF EXECUTIVE OFFICE

1. Introduction

Background

1.1. Pharmacy practice is growing and with the COVID-19 pandemic, new ways of conducting the pharmacy practice also came into play, including the need for internet pharmacy.

1.2. The illegal sale of human medicinal products to the public online is a major threat to public health as falsified medicinal products may reach the public in this way.

1.3. PPB has developed a guideline on internet pharmacy services to enhance safety and provide greater assurances to the public regarding the provision of internet pharmacy services from registered pharmacy premises in Kenya.

1.4. The guidance in this document is expected of owners and superintendents of registered pharmacy premises in Kenya to meet in the provision of internet pharmacy services.

1.5. This guideline considers all aspects of the legal framework around medicines and pharmacy practice under CAP 244 Laws of Kenya

1.6. An internet pharmacy service should not be a substitute for a face-to-face consultation with a pharmacist or other appropriate healthcare professional, but when used safely and appropriately, online access to medicines and healthcare advice can offer benefits to patients.

1.7. A registered pharmacy which offers to sell or supply medicines (or other pharmaceutical products) and/or provides other professional services over the internet or makes arrangements for the supply of such products or provision of such services over the internet must comply with this guideline.

1.8. This document expands on the principles of pharmacy practice to explain the pharmacists and pharmaceutical technologists' professional responsibilities when selling and supplying medicines via the internet.

Legal framework

1.9. The Pharmacy and Poisons Board (PPB) is the national regulatory authority established under the Pharmacy and Poisons Act, Cap 244 Laws of Kenya ("the Act").

1.10. The Act mandates the PPB to regulate medical products, health technologies and the profession of Pharmacy. Sec 3B (2)(j) of the law provides for the Board to carry out the function of inspecting and licensing all manufacturing premises, importing, and exporting agents, wholesalers, distributors, pharmacies, including those in hospitals and clinics, and other retail outlets.

1.11. The Pharmacy and Poisons (Amendment) Rules 2022 empowers the Board to license online pharmacies.

Scope

1.12. This guideline is applicable to all premises that carry out the sale of Health Products and Technologies over the internet in Kenya.

2. Requirements for Internet Pharmacy Services in Kenya

Registration of Internet pharmacy services

2.1. All pharmacies in Kenya intending to sell medicines online to the general public shall be registered with PPB and be on the list of registered online retail sellers in Kenya.

2.2. No person/pharmacy shall operate an online pharmacy in Kenya without the explicit authorization of the Board.

2.3. The pharmacies shall display on every page of their website offering medicines for sale, the health safety code provided by the PPB.

2.4. Pharmaceutical services provided to the public via the internet include amongst other things, the dispensing of prescriptions, the sale of medicines and the provision of information on website pages.

2.5. A person carrying on an internet pharmacy business shall be taken to be a person lawfully conducting such a business, that is, a qualified pharmacist or pharmaceutical technologist owner or if the pharmacy is owned by a 'body corporate' (for example a company) a superintendent pharmacist or pharmaceutical technologist

2.6. A pharmacy offering internet services shall meet the following:

2.6.1. There shall be pharmacy premises with a permanent physical address, from where the sale and supply of medicines and medical products takes place, that meet the requirements and is duly registered by the Board.

2.6.2. The pharmacy shall have a superintendent pharmacist or pharmaceutical technologist registered with the board

2.6.3. In addition to the requirements of setting up a retail premise *(Guidelines for Registration and Licensing of Premises in Kenya PRA/LPP/GUD/060)* the following will be required:

a) Reasonably secure encryption with valid licenses

b) Any individual buying medicines online should be able to check if the website is legitimately registered and will be able to check the health safety code for verification.

2.6.4. There shall be no advertisement/promotion of Prescription Only Medicines to the general public

2.6.5. Internet site shall be set up such that there shall be pages that can only be accessible to healthcare workers with adequate controls to ensure that the pages are not accessed by the general public

2.6.6. The superintendent pharmacist or pharmaceutical technologist shall be responsible for ensuring regulatory compliance with this guideline.

3. Minimum Standards for Pharmacies Operating Internet Sites

3.1. Internet websites that sell medicine directly to the public must be operated by and constitute part of a pharmacy licensed by the board.

3.2. Such a website must fall under the authority of the responsible pharmacist of the pharmacy of which it forms a part and be operated in compliance with all relevant legislation including CAP 244 and all other relevant guidelines and regulations.

3.3. An Internet site cannot exist/operate independently of a pharmacy.

3.4. The following standards apply—

3.4.1. The decision to sell a medicine must be made by a pharmacist who must be available for consultation with clients.

3.4.2. The handling of prescriptions must follow all legislation that applies to the supply of medicine including that—

(a) a pharmacist must verify the authenticity of all electronic prescriptions.

(b) a permanent copy of all electronic prescriptions must be recorded and filed.

(c) all electronic prescriptions must be followed by the original prescription within seven working days.

(d) only the prescriptions of persons authorized to prescribe in Kenya

may be dispensed.

(e) the website must be operated in compliance with all regulations and rules regarding the system for medicine and controlled substances and the advertising and promotion of medicine.

3.4.3. The home page of any pharmacy Internet site must identify the website as being operated by a pharmacy licensed by the board and must provide at least the following information:

(a) the health safety code registration number of the pharmacy.

(b) the physical and postal addresses of the pharmacy.

(c) telephone number(s) to contact the pharmacy.

(d) the name of the responsible pharmacist.

(e) the hours that a pharmacist is available for consultation.

3.5. Patient health information must be collected, recorded, and used in a manner that protects confidentiality and privacy.

3.6. The Internet site must provide information to the public regarding policies and procedures for the following:

(a) counseling patients on new and repeat prescriptions.

(b) counseling patients on medicines sold without a prescription.

(c) delivery of medicine.

(d) return of medicines.

(e) complaints procedures.

(f) confidentiality of patient information.

(g) hours of provision of service or hours of business of the pharmacy.

3.7. The responsible pharmacist shall inform the Board of any information as required in terms of the CAP 244 as well as the following:

(a) when a pharmacy is going to be conducting business over the Internet.

(b) the Internet address.

(c) nature of the business (e.g., Rx, OTC or both, specialties, etc.).

(d) the telephone number if different from the pharmacy.

3.8. The Internet operation must be available for onsite inspection by PPB inspectors.

3.9. Pharmacists remain professionally liable regardless of any disclaimers which may be posted on the website.

3.10. Appropriate records shall be kept for all procedures undertaken and

3.11. the pharmacy procedures shall be recorded in writing and/or electronic form.

3.12. The pharmacy procedures shall be made available to the Board's inspectors during inspection.

3.13. The online pharmacy records shall be preserved for a period of not less than five years commencing on:

a) in the case of a record in electronic form, the day on which it is created

b) in the case of a written record, the last day to which the record relates.

3.14. The superintendent shall ensure that there is an in-built mechanism or alarm to alert and thereby restrict excessive/multiple orders of medicines.

3.15. Prescriptions for Controlled Drugs (CDs) **shall not be supplied** via the internet. (Consider additional safeguards for others such as antibiotics, medicines that require close monitoring e.g., with narrow therapeutic index (lithium, warfarin, sodium valproate); drugs that can be used for abortion, beauty enhancement/enlargement etc.)

3.16. The seller of the medicine should only source medicines from authorized manufacturers, importers, or distributors in Kenya.

4. Professional Services

4.1. Patients shall be entitled to the same quality of pharmaceutical care irrespective of whether the service is provided on-line or face-to-face on the pharmacy premises.

5. Dispensing

5.1. When selling or supplying medicines via the internet, the pharmacist or pharmaceutical technologist shall:

5.1.1. Ensure that only PPB-registered and retained medicines are sold/dispensed to clients

5.1.2. Ensure that for prescription-only medicine there's a valid prescription as per Cap 244 requirements.

5.1.3. For any over-the-counter medicine, the practitioner shall acquire all the relevant patient information and rationalize the request to purchase medicines.

5.1.4. Ensure that advice is available to all prospective purchasers of medicines. This is in addition to and/or to supplement the information contained in the PIL (Patient Information Leaflet).

5.1.5. Establish that the intended user or the guardian is the person requesting the product: sufficient information about the patient and the condition(s) being treated shall be obtained.

5.1.6. Assess the suitability and safety of the product for the intended user and ensure that it is appropriate for the reported indication.

5.1.7. Provide appropriate counseling or advice on the safe and effective use, storage, and disposal of the product to be supplied. The patient should be provided with a contact telephone number on which they can access appropriate

information and advice from a suitably trained professional.

5.1.8. Be aware of the potential abuse of some medicines and other products. The pharmacist or pharmaceutical technologist shall have measures in place to identify requests for large quantities of a product, or abnormally frequent requests and thereupon refuse to make a supply where there are reasonable grounds for suspecting misuse and/or abuse.

5.1.9. Advise the patient to consult a local pharmacy or other appropriate healthcare professional whenever a request for a medicine or the symptoms described indicate that the patient's best interests would be served by a face-to-face consultation.

5.1.10. Inform the patient of the identity of the pharmacist or pharmaceutical technologist assuming professional responsibility for the supply of medicines

5.1.11. Verify the authenticity and validity of the prescription.

6. Information and advice

6.1. When providing internet pharmacy services, the pharmacist or pharmaceutical technologist must ensure that:

6.1.1. Generic healthcare advice (i.e., not specific to the patient) provided on pharmacy websites is accurate, up-to-date, and presented in a professional manner.

6.1.2. All information relating to specific products complies with the marketing authorization, the patient information leaflet (PIL), the Board's guideline and other relevant legislative requirements.

6.1.3. Information relating to medicines includes all relevant details of contraindications and side-effects.

6.1.4. Product recommendations are given only in respect of individual patients.

6.1.5. Any advertising or publicity complies with relevant Board's Guidelines for Advertisement and Promotion of Health Products and Technologies (HPT/PDS/GUD/046) and legislations (The Pharmacy and Poisons (Amendment) Rules, 2022).

6.1.6. Any advice and/or written information is provided to the patient in English or Swahili.

6.2. The pharmacist should advise the patient to consult a local pharmacy or other appropriate healthcare professional whenever a request for a medicine or the symptoms described indicate that the patient's best interests would be served by a face-to-face consultation.

6.3. The pharmacist should inform the patient of the identity of the pharmacist assuming professional responsibility for the supply of medicines.

7. Delivery of medicines

7.1. When delivering medicines to a patient, the superintendent shall:

7.1.1. Ensure that delivery mechanisms safeguard confidential information about the individual patient's medication.

7.1.2. Take adequate steps to ensure that the delivery mechanism used is secure and that medicines are delivered to the intended user promptly, safely and in a condition appropriate for use.

7.1.3. Ensure the integrity of cold chain and safe transportation of liquid or other vulnerable packaging.

7.1.4. Ensure medicines are packed, transported, and delivered in such a way that their integrity, quality, and effectiveness are preserved. Care must be exercised with thermo-labile or photo sensitive products.

7.1.5. Ensure that the delivery mechanism used provides a verifiable audit trail for the medicine from the initial request for a medicine through to its delivery to the patient, or its return to the pharmacy in the event of a delivery failure.

7.1.6. Returned medicines should not be resold. There shall be a procedure for handling such returned medicines.

7.1.7. Ensure that there is a robust and reliable system to undertake a recall of medicines incase a need arises.

8. Record keeping

8.1. The pharmacist or pharmaceutical technologist must maintain records about online consultations and medicines supplied which are sufficient to guard against risks of abuse or misuse.

8.2. A verifiable audit trail from the initial request for a medicine through to its delivery to the patient shall exist. These records may be subject to audit / inspection.

8.3. A pharmacist or pharmaceutical technologist providing internet pharmacy services shall maintain records of:

8.3.1. The identity of customers (i.e., name and address) who have been supplied with medicines via the internet.

8.3.2. Details of the medicines including quantities requested and supplied.

8.3.3. Details of any consultation with the patient or prescriber, interventions made, and/or advice given.

8.3.4. The information upon which decisions to supply were made.

8.3.5. The identity of the pharmacist or pharmaceutical technologist who has assumed professional responsibility for the supply of medicine following an online request to purchase.

8.4. All relevant records must be maintained for a period of not less than 5 years.

9. Security and confidentiality

9.1. All patient data and handling of the same shall comply with the Data Protection Act of 2019

9.2. The confidentiality and integrity of all patient information shall be protected to the standard specified by the International Organization for Standardization (ISO) in ISO/ IEC 27001

9.3. All patient data transmissions shall be encrypted to prevent the possibility of the internet service provider or any other unauthorized party accessing patient information either accidentally or deliberately.

9.4. Patient information shall be backed up daily and kept in a secure, fire-proof system including offsite back up.

9.5. Any requests for information on a patient's medicines must only be made by either the patient or an agent acting on his behalf and be fully verifiable.

10. Website requirements

10.1. Patients must be able to easily identify who is operating an internet site from a registered pharmacy premises.

10.2. Pharmacy websites must clearly and conspicuously display:

10.2.1. The Health Safety code of the premise;

10.2.2. The address of the registered pharmacy premises at which the business is conducted, as well as, the premises registration number, email address, and telephone number.

10.2.3. The name and registration number of the superintendent Pharmacist in the documentation supplied to the patient.

10.2.4. Details of how to make a complaint about the online services provided;

10.2.5. An Extended Validation SSL (EV-SSL) certificate indicates to users that the website is secure and encrypted in terms of an individual entering any sensitive information.

10.3. The website that is used to sell medicines online must also contain the contact details of the PPB and a hyperlink to the PPB website. This information need only appear once on the website. The following contact details and website address should be used:

The Pharmacy and Poisons Board,
Lenana Road Opposite Russian Embassy,
P. O. Box 27663- 00506, Nairobi, Kenya
Tel: +254 709 770 100
Website: www.pharmacyboardkenya.org.ke
Email: info@pharmacyboardkenya.org.ke

11. Technology Equipment and Software

11.1. The pharmacy shall have validated, and fit-for-purpose technology equipment and software needed to provide online pharmacy services. Examples include dispensing systems, labelling equipment and mobile devices used for remote access.

11.2. There shall be plans on how to manage the risk of IT equipment and software failure and disruptions.

11.3. IT equipment shall:

11.3.1. meet the latest security specifications and the security of data should be protected when in transit, by either wired or wireless networks.

11.3.2. be calibrated, maintained, and serviced regularly in line with the developer's specifications

11.4. Software and operating systems shall:

11.4.1. be robust enough to handle the volume of work

11.4.2. have control systems built in to help manage risk, such as alarms or alerts for:

(a) Orders for controlled medicines

(b) Suspicious, excessive, or multiple orders of medicine

11.4.3. keep records of all decisions made, and dispensing records for all medicines and medical products (refer above).

12. Quality Assurance

12.1. The management of the online pharmacy shall make regular audits of the following:

(a) Personnel: Staffing levels, skills, and training gaps

(b) communication systems between staff, prescribers, other healthcare workers, patients, and clients

(c) quality of professional services

(d) systems and procedures for receiving prescriptions

(e) systems and procedures for dispensing prescriptions

(f) systems and procedures for secure posting and delivering medicines and other products

(g) documentation and records

(h) Validation of IT equipment and software

(i) security and confidentiality

(j) Information on the website

(k) Customer feedback

(l) Customer complaints

12.2. Audit schedules and reports must be availed to the PPB inspectors when required

13. Requirements for Online Pharmacy (ICT)

13.1. Registration as Data Controller under Data Protection Act 2019

The online pharmacy shall register as a data controller with the Office of the Data Protection Commissioner (ODPC) as per section 18 of the Data Protection Act, 2019, and Data Protection (Registration of Data Controllers and Data Processors) Regulations, 2021. Registration certificate from the ODPC shall be a minimum requirement

13.2. Data Processing

The online pharmacy shall be required to **process data lawfully**; minimize **the collection of data; restricts further processing of data**; require data controllers and processors to ensure data quality, and **establish and maintain security safeguards to protect personal data**

In this regard, the following shall be required:

a) Only collect information that is permitted under the data protection act 2019 and regulations 2021 for personal information

b) Store data in locations as designated by the data protection act 2019 and regulations 2021 on data centers and servers

c) Provide security for data at rest and data in transit as per the data protection act 2019 and regulations 2021.

13.3. Rights of the Subjects

The online pharmacy must ensure all subjects' rights are observed per section 26 of the Data Protection Act, 2019, and Data Protection (Registration of Data Controllers and Data Processors) Regulations, 2021.

13.4. Duties of the online pharmacy

Before and after collecting data, the online pharmacy shall inform the subjects of their rights and collect and manage the data as per section 26 of the Data Protection Act, 2019, and Data Protection (Registration of Data Controllers and Data Processors) Regulations, 2021.

13.5. Requirements for Registration at PPB

The following details shall be provided to the board for purposes of registration.

a) Certificate of registration as a data controller from the ODPC.

b) Contract agreement or proof of ownership of data center located within Kenya.

c) Valid X.509 public key certificate recognized under Kenya Information and Communications Act, 1998, the Kenya Information and Communications (Electronic Certification and Domain Name Administration) Regulations, 2010 for the online pharmacy domain. Certificates issued by foreign certificate authorities must meet the same conditions as locally issued certificates.

d) Online pharmacy link for public access. For mobile applications, Application Programming Interface (API) links must be provided and should be accessible. For testing and monitoring purposes, the online pharmacy shall provide user-level read-only credentials to PPB. All links should be accessible through open-source REST client tools like web browsers.

e) Online pharmacies using third-party platforms shall attach contract and service level agreements showing the responsibilities of each party.

13.6. Requirements for Renewal

The following details shall be provided to the board for purposes of license renewal.

a) Annual renewal certificate as a data controller from the ODPC.

b) Contract agreement or proof of ownership of data center located within Kenya.

c) Valid X.509 public key certificate recognized under Kenya Information and Communications Act, 1998, the Kenya Information and Communications (Electronic Certification and

Domain Name Administration) Regulations, 2010 for the online pharmacy domain. Certificates issued by foreign certificate authorities must meet the same conditions as locally issued certificates.

d) Online pharmacy link for public access. For mobile applications, Application Programming Interface (API) links must be provided and should be accessible. For testing and monitoring purposes, the online pharmacy shall provide user-level read-only credentials to PPB. All links should be accessible through open-source REST client tools like web browsers.

e) Online pharmacies using third-party platforms shall attach contract and service level agreements showing the responsibilities of each party.

14. Suspension or Revocation of Internet Pharmacy License

The Board has the power to suspend or revoke the online pharmacy license if it has been closed or if the conditions under which the license was issued are no longer being met.

References

1. PPB Guidelines on Good Distribution Practices for Medical Products and Health Technologies in Kenya (HPT/ISE/EFS/GUD/019)
2. PPB Guidelines for Registration and Licensing of Premises (PRA/LPP/GUD/060)
3. Standards and Guidance for Internet Pharmacy Services in Northern Ireland
4. MHRA guidance. Selling human medicines online (distance selling) to the public.

Revision History

Revision No:	Date	Sections Revised	Description of change
01	18-11-2022	General	General editing and harmonization of terminologies with other PPB documents:<br>• health products to replace "medical products"
		1.11	Added to legal framework 1.11 The Pharmacy and Poisons (Amendment Rules) 2022 empowers the Board to license online pharmacies
		1.12	This guideline is applicable to all premises that carry out the sale of Health Products and Technologies over the internet in Kenya
		2.6.6.	The superintendent pharmacist or pharmaceutical technologist shall be responsible for ensuring regulatory compliance with this guideline.
		13.	Added: Requirements for online Pharmacy (ICT) under the following sub-headings<br>13.1. Registration as Data Controller under Data Protection Act 2019<br>13.2. Data Processing<br>13.3. Rights of the Subjects<br>13.4. Duties of the online pharmacy<br>13.5. Requirements for Registration at PPB<br>13.6. Requirements for Renewal
		14.	Suspension or Revocation of Internet Pharmacy License:<br>The Board has the power to suspend or revoke the online pharmacy license if it has been closed or if the conditions under which the license was issued are no longer being met.

The Pharmacy and Poisons Board

P. O. Box 27663- 00506 Lenana Road Opposite Russian Embassy Nairobi,

Tel: +254 709 770 100

Website: www.pharmacyboardkenya.org.ke

Email: info@pharmacyboardkenya.org.ke
